# The Latest Advances in Mechanically Robust Self‐Healing Polyurea Based on Dynamic Chemistry

**DOI:** 10.1002/advs.202414788

**Published:** 2025-04-17

**Authors:** Yujia Hao, Guangming Zhu

**Affiliations:** ^1^ School of Chemistry and Chemical Engineering Northwestern Polytechnical University Xi'an 710129 China

**Keywords:** dynamic chemistry, mechanical performance, polyureas, self‐healing

## Abstract

Polyureas are widely used in many fields such as civil, industry, and defense due to their excellent performance and structural adjustable properties. The development of self‐healing polyurea materials with high strength and toughness, key connotations of their advanced applications, is both fascinating and challenging because these properties are associated with conflicting structural features, making it difficult to optimize these contradictory properties in a single material. In this review, the relationship between polyurea structure and performance is discussed, and the design strategy of self‐healing polyurea networks based on dynamic interactions that allow for balancing high mechanical performance and repairability is delineated from a molecular design point of view. Lastly, a summary of the potential applications of polyurea in the fields of sensing, protective coatings, and recycling, as well as possible future challenges, is presented.

## Graphical abstract

Solving the contradiction between strength, toughness, and self‐healing properties is extremely important to realize the breakthrough of polyurea applications. This review summarizes and discusses common strategies for the preparation of high‐performance and self‐healing polyurea materials based on dynamic chemistry,  providing molecular‐level insights for polyurea design. Furthermore, the paper also presents application scenarios for such materials and current challenges in the field, providing guidance for future developments.

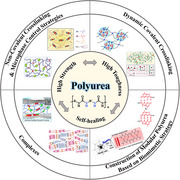

## 1 Introduction

Polyurea refers to an important class of polymer materials containing urea bonds.^[^ ^1^ ^]^ By adjusting the structure and formulation, polyurea can exhibit a wide range of mechanical properties, from soft rubber to hard plastic.^[^ ^2^ ^]^ Excellent physicochemical properties and structural tunability have led to the use of polyureas in a wide variety of industrial applications, including adhesives, coatings, foams, textiles, and more.^[^ ^3^, ^4^, ^5^ ^]^ In particular, mechanically robust polyureas (tensile strength >20 MPa, toughness >100 MJ·m^−3^) are critical to both traditional engineering and cutting‐edge research fields, such as corrosion protection, ballistic protection, and blast resistance.^[^ ^6^, ^7^ ^]^ Unfortunately, although these two properties may seem similar to many, changes in the structure of material usually impact strength and toughness in quite distinct ways.^[^ ^8^ ^]^ Strength in a material usually implies resistance to plastic deformation, defined in uniaxial tension, compression, or bending, either at first yield (yield strength) or at maximum load (ultimate strength), whereas toughness typically indicates resistance to fracture, measured by the energy required to cause fracture.^[^ ^9^ ^]^ From the microscopic perspective, the high strength of polymers is associated with strong directional bonding, whereby the molecular chain segments are anchored in such a way that they do not migrate easily and plastic deformation is limited. Correspondingly, the size of the plastic deformation zone in front of the crack tip to dissipate local stress is also limited, leading to a decrease in the toughness of the material.^[^ ^8^, ^10^, ^11^ ^]^ From this, it appears that strength and toughness are typically mutually exclusive, so designing strong and tough polyurea materials is inevitably a compromise.^[^ ^12^ ^]^

Since polyurea is typically used in applications that do not allow catastrophic fracture, for example, various types of protective coatings for corrosion and penetration,^[^ ^13^ ^]^ impact,^[^ ^14^ ^]^ and ballistic protection,^[^ ^15^ ^]^ medical implants like scaffolds for human tissues,^[^ ^16^ ^]^ and wearable devices like electronic skins,^[^ ^17^ ^]^ it can be argued that toughness is more important than strength. Following the theories associated with fracture mechanics, toughening strategies for materials are categorized into intrinsic and extrinsic approaches.^[^ ^8^ ^]^ Enlarging the plastic zone by changing the nature, distribution, and/or interfacial properties of the second‐phase particles to inhibit damage in the form of microcracking or microvoid formation ahead of the crack tip is referred to as intrinsic toughening. Reducing the crack driving force actually experienced at the crack tip by microstructural mechanisms that act behind the crack tip, such as crack bridging and in‐situ phase transitions, is known as extrinsic toughening.^[^ ^8^, ^11^, ^18^ ^]^ Intrinsic toughening is the main source of fracture resistance in ductile materials like polymers, which origins often on the smaller sub‐micrometer length scales.^[^ ^11^ ^]^ A number of specialized biological and natural materials have shown an amazing balance of strength and toughness by judiciously combining these toughening mechanisms, which has given researchers some inspiration. The common aspect of these materials is that they exhibit hierarchical structure, with unique structural features at multiple length scales from the molecular to the near‐macroscopic dimensions.^[^ ^10^, ^19^, ^20^, ^21^ ^]^ Nacre in nature, for example, has a “brick‐and‐mortar” structure, with the “bricks” consisting of mineral aragonite platelets with a length of 5 to 10 µm and a thickness of 0.5 µm, separated by an organic biopolymer “mortar”.^[^ ^22^ ^]^ The “bricks” are responsible for the high strength, while the “mortar” acts as a lubricant to allow the movement of the aragonite platelets to relieve the high local stresses; this constitutes the intrinsic toughening mechanism and endows the nacre with high strength and toughness.^[^ ^10^, ^11^ ^]^ Fortunately, the intrinsic structure of polyurea soft and hard inserts makes it not difficult to mimic hierarchical structures to achieve toughening. Several studies have modulated the microphase separation of polyurea by molecular‐level design, so that the polymer network combines both rigid network and soft matrix as well as hierarchical interactions, which ultimately achieves a combination of high mechanical properties. For example, Wang et al. prepared two polyurea elastomers with high mechanical strength (36.93 MPa and 37.78 MPa) and high toughness (230.78 MJ·m^−3^ and 156.29 MJ·m^−3^) by introducing the rigid benzene ring structure between the soft and hard segments.^[^ ^23^ ^]^ Both polyureas structures are characterized by the microphase separation in which the hard segment units are crosslinked by reversible hydrogen bonding, while the rigid benzene ring structure promotes the formation of hard domains. Niu et al. developed a rather tough polyurea‐urethane material by introducing weak hydrogen bonds, strong hydrogen bonds, and dynamic silyl ether covalent bonds.^[^ ^24^ ^]^ The multilevel and intensive dynamic interaction not only achieves reinforcement but also provides large energy dissipation, which ultimately contributes to the high toughness (256 MJ·m^−3^) and strength (73 MPa) of the material. The emergence of these high‐performance polyurea materials has greatly broadened the scope of application of polyurea materials, especially suitable for national defense, engineering and other fields that have very high requirements for material performance.

As mentioned above, polyurea has many excellent properties and its mechanical properties can be customized to the specific application environment. However, even though many high‐performance polyurea materials have been designed for their durability, the materials are still inevitably subject to mechanical/chemical damage during actual application.^[^ ^25^ ^]^ Unfortunately, most robust polyurea materials lack intrinsic healing dynamics, making it difficult to repair damage and recycle. As a result, these materials are discarded upon failure, resulting in serious environmental pollution and resource waste.^[^ ^26^ ^]^ Integrating self‐healing ability into polyurea materials is considered a promising approach to extending their service life and reducing raw material consumption and environmental pollution.^[^ ^27^ ^]^ The emergence of dynamic chemistry has greatly contributed to the development of self‐healing polymers, including polyureas. Dynamic chemistry is based on dynamic covalent bonds (such as Diels‐Alder bonds, imine bonds, disulfide bonds) or dynamic non‐covalent interactions (including hydrogen bonds, metal coordinates, and ionic bonds) which are capable of reversible exchange or breakage/reorganization under certain external stimuli (e.g., heat, light, solvent).^[^ ^28^, ^29^ ^]^ After introducing dynamic chemistry into polymers, by means of reversible chemical linkages between interconnected monomer units, polymers are able to achieve self‐healing by building adaptable networks that respond dynamically to stimuli. These dynamic interactions make polyurea highly versatile and durable materials for advanced applications. Although significant progress has been made in self‐healing polymer materials including polyurea based on dynamic chemistry, over the past decade, obtaining self‐healing polyurea materials that are mechanically robust and maintain excellent dynamic properties remains a major challenge in the field of polymer science because of several inherent trade‐offs between self‐healing and mechanical robustness.

Self‐healing process is governed by thermodynamics and/or reaction kinetics. In the case of damage to the polymer network, chain cleavage and/or chain slippage mainly occur.^[^ ^27^ ^]^ The former generates reactive groups, while the latter may lead to conformational changes in the polymer network. Both dynamic interactions and mobility of the polymer chains are essential to enable the repair of the ruptured network. Dynamic interactions allow for the possibility of reconnection of fractured polymer chains, and chain mobility facilitates the contact of reactive groups through chain rearrangement, where bond reorganization occurs and the conformation of the polymer network is restored, leading to further restoration of mechanical properties.^[^ ^30^ ^]^ However, the high chain stiffness, chain entanglement, and strong bonding required for mechanically robust network conflict with the high mobility and dynamic exchange required for damage repair. Simultaneously realizing the highly robust mechanical properties and efficient self‐healing of polyureas through molecular structure design is difficult but an attraction in this field. Conventional design strategies are no longer satisfactory and researchers have attempted to solve this problem by searching for new dynamic motifs or dynamic chemical strategies. For example, by mimicking one of nature's strongest materials, spider silk, which has a high density of hydrogen bonds, the researchers introduced acylsemicarbazide (ASCZ), a modular dynamic motif, into the polyurea network to develop mechanically tough and effectively damage‐healing polyurea materials that are mechanically tough and can effectively repair damage.^[^ ^31^, ^32^ ^]^ By constructing multi‐level hydrogen bonds with different strengths and densities,^[^ ^33^, ^34^ ^]^ combining multiple dynamic interactions,^[^ ^24^, ^35^ ^]^ building double/multiple crosslinked networks,^[^ ^36^, ^37^ ^]^ etc., an effective balance between mechanical and self‐healing properties can also be realized. Previous studies have shown that the formation of nano‐ to micrometer‐scale microphase separations will facilitate polymer self‐healing.^[^ ^38^ ^]^ As a result, a significant portion of the research has focused on the modulation of the microphase‐separated structure of polyureas, which extends to a series of strategies including dynamic enhancement of hard domains,^[^ ^39^, ^40^ ^]^ hard phase‐locking strategies,^[^ ^40^, ^41^ ^]^ the construction of asymmetric/loosely stacked hard domains,^[^ ^42^, ^43^ ^]^ and the construction of rigid hard domains to trigger strain‐induced crystallization to achieve self‐enhancement.^[^ ^44^, ^45^ ^]^ These strategies not only solve the performance contradictions of traditional polyurea materials, but also further push the boundaries of polymer science, and open up new possibilities for sustainable materials with extended service life and reduced environmental impact. The multifunctionality, sustainability and wide range of application prospects brought about make polyurea one of the current research hotspots in the field of materials.

**Figure 1:**
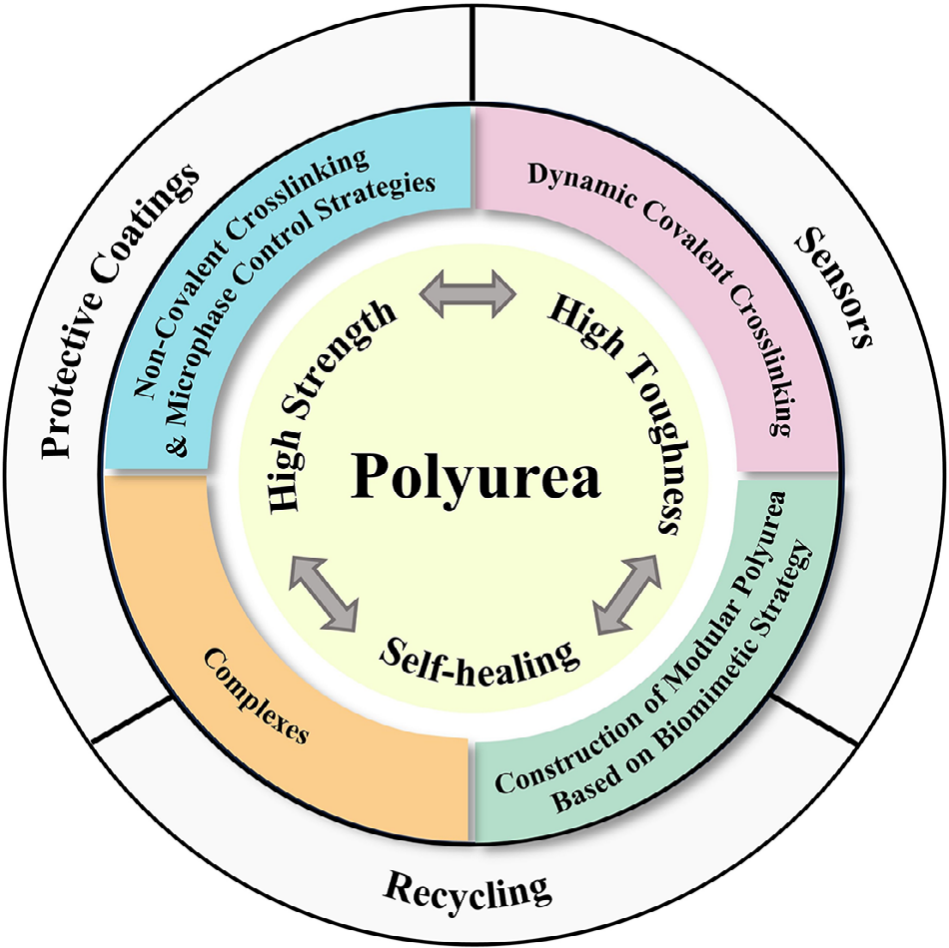
Schematic representation of the synthetic strategies and various applications of high‐performance self‐healing polyurea.

The excellent performances of polyureas are generally attributed to their unique chemical structure and understanding the structure‐property relationships is critical in the development and application of high‐performance self‐healing polyurea materials. This review first introduces the chemical synthesis and structural characteristics of polyurea; the factors affecting the performance of polyurea are analyzed from the perspective of molecular design, including isocyanate, chain extender, soft chain segment structure, and so on. To date, several review papers have focused on the various types of self‐healing chemistries and methods used to synthesize self‐healing polyureas.^[^ ^46^, ^47^, ^48^, ^49^, ^50^, ^51^ ^]^ As far as we know, there are very few reports that exclusively summarize how to achieve a compromise between high mechanical properties and self‐healing properties. In this review, we list a selection of mechanically robust self‐healing polyurea systems synthesized by researchers over the past few years and summarize the methods from a chemistry perspective, as outlined in **Figure** **1**. In addition, this paper will discuss directions for the application of high‐performance self‐healing polyurea materials, challenges encountered during the preparation of the materials, as well as recommendations for future research in this area.

## 2 Structure–Property Relationships

**Figure 2:**
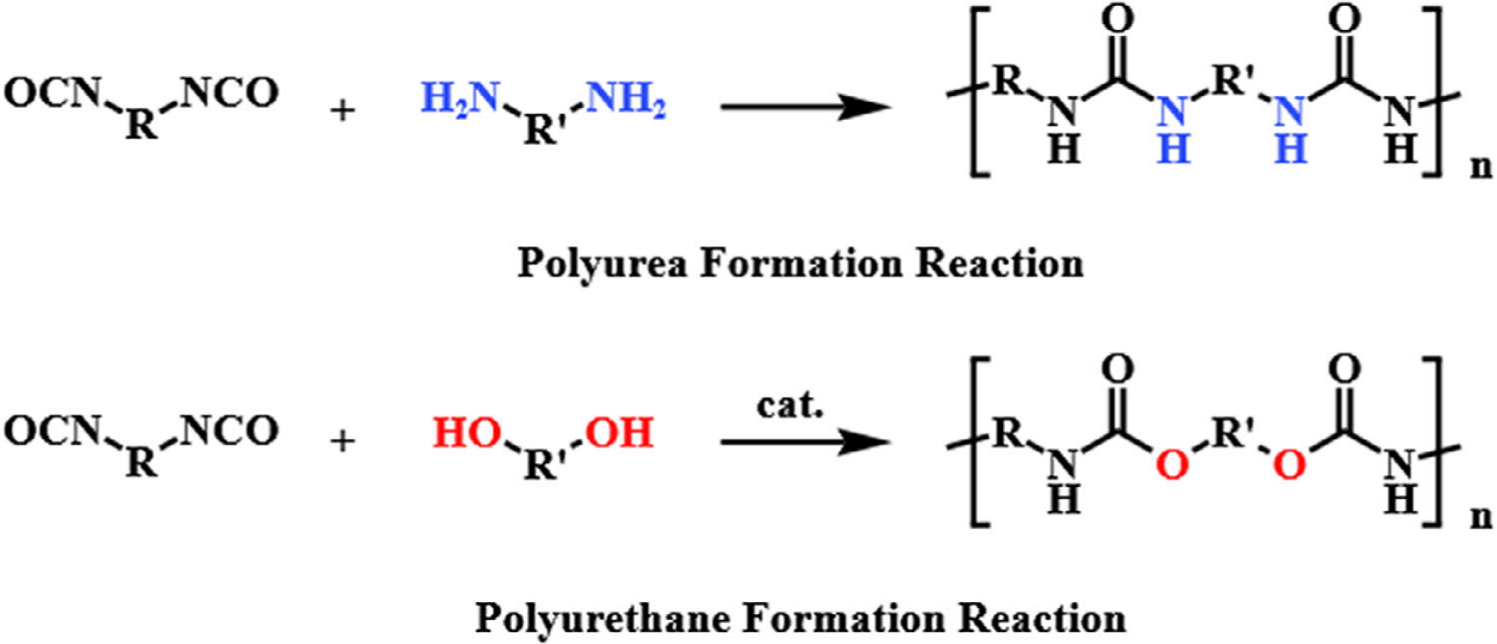
Polyurea and polyurethane formation reaction.

Polyurea is a block copolymer synthesized from isocyanate prepolymer and amine‐terminated chain extender. Polyurea was first mentioned in the literature in 1948, but only really gained commercial attention in the late 1980s, due to the development of spray polyurea elastomer technology.^[^ ^52^ ^]^ Polyurea is an extension of polyurethane chemistry. A simple illustration of the “polyurea” and “polyurethane” reaction is given in **Figure** **2**. The main distinguishing between the polyurea over polyurethane is that amine‐terminated (‐NH_2_) chain extenders are used rather than hydroxyl‐terminated (─OH) chain extenders.^[^ ^53^ ^]^ During polyurea synthesis, soft segment resins, including amine‐terminated or hydroxyl‐terminated polymer resins, are commonly used to prepare isocyanate prepolymers or quasi‐prepolymers by reacting with isocyanates.^[^ ^54^ ^]^ Specifically, the polyurea synthesized with isocyanate, amine‐terminated resin, and amine‐terminated chain extender is usually considered to be pure polyurea, and the polyurea synthesized from isocyanate, hydroxyl‐terminated resin, and amine‐terminated chain extender is referred to as polyurea‐urethane.^[^ ^53^ ^]^ In an early study, Born and Hespe found that the urea group is more polar than the urethane group and can form strong bifurcated hydrogen bonds, resulting in a polyurea structure with stronger interchain interactions and a more pronounced microphase separation.^[^ ^55^ ^]^ This explains the generally higher thermal stability and superior mechanical properties of polyureas compared to polyurethanes.

Due to the differences in the thermodynamic properties of the soft and hard segments in polyurea, as well as hydrogen bonding as the main kinetic factor, the soft and hard segments separate from each other, forming their own independent micro‐regions on a microscopic level. The nanoscale hard domains aggregated from the hard segments are dispersed in a continuous soft matrix composed of soft segments.^[^ ^56^, ^57^ ^]^ This phenomenon is called “microphase separation” and is a typical structural feature of polyurea, in which the hard domains serve as crosslinking and reinforcement, while the soft domains play a role in toughening.^[^ ^1^, ^58^ ^]^ If hydrogen bonding can only be formed within the microstructural domains of the hard segments, the degree of microphase separation is increased; conversely, microphase mixing is caused if partial hydrogen bonding can also be formed between hard and soft segments.^[^ ^59^ ^]^ Research on the microstructure of the material shows that structure, crystallinity, polarity, symmetry, and hydrogen bonding interactions of the soft and hard segments all significantly affect the microphase structure and the degree of microphase separation of polyurea, which in turn affects the mechanical and self‐healing properties of the material.^[^ ^48^, ^60^ ^]^ The properties of a material are determined by its micromorphology, which is directly derived from its chemical composition. Therefore, many contradictions between mechanical and self‐healing properties need to be resolved by first understanding the relationship between the structure and properties of molecules such as isocyanates, soft segment resins, and chain extenders.

### 2.1 Isocyanate

Isocyanate is the key raw material in the production of polyurea, and its chemical structure plays an important role in the mechanical and self‐healing properties of polyurea. Isocyanates commonly used in this field and their structures are as follows: 4,4′‐diphenylmethane diisocyanate (MDI), toluene diisocyanate (TDI), hexamethylene diisocyanate (HDI), 4,4′‐Methylenebis(cyclohexyl isocyanate) (HMDI), isophorone diisocyanate (IPDI).^[^ ^61^ ^]^ Aromatic isocyanates are more reactive than aliphatic isocyanates. The use of rigid aromatic isocyanates is favorable for improving the mechanical strength of the product, but may reduce chain mobility, thus affecting the toughness and self‐healing effect.^[^ ^53^, ^62^, ^63^, ^64^ ^]^ IPDI and HMDI containing alicyclic structures are commonly used to design and prepare high‐performance self‐healing polyurea materials. The presence of the alicyclic ring can make the hydrogen bonds loose stacking in the hard domains, which is conducive to the exchange of hydrogen bonds and other dynamic bonds.^[^ ^32^, ^65^, ^66^ ^]^ This not only facilitates energy dissipate and improves the toughness of the material, but also confers good self‐healing properties.^[^ ^32^ ^]^ Besides, the symmetry of the isocyanate has a great impact on the morphology and properties of the resulting polyurea. Polyureas based on symmetric isocyanate usually exhibited improved mechanical properties.^[^ ^67^, ^68^, ^69^ ^]^ Moreover, hexamethylene diisocyanate trimer (Tri‐HDI) is usually introduced as the covalent crosslinking agent in the preparation of polyureas.^[^ ^70^, ^71^, ^72^ ^]^ The six‐membered ring of tri‐HDI, a quite stable structure that can provide the polyurea network with a tough skeleton. Moderate chemical cross‐linking not only enhances the mechanical strength of the material, but also reduces the creep of macromolecules and improves the elastic recovery ability of the material.^[^ ^72^ ^]^

### 2.2 Soft Segment

Soft segment resins commonly used in polyurea production typically include polypropylene glycol (PPG), polycaprolactone (PCL), polytetrahydrofuran (PTMEG), polydimethylsiloxane (PDMS), polycarbonate (PC), etc. of various molecular weights, either amino‐ or hydroxyl‐ terminated.^[^ ^73^, ^74^ ^]^ The soft segments present high flexibility, which facilitates the toughening of polyurea and makes a large contribution to self‐healing. According to thermodynamics, different types of soft segments have different solubility parameters, affecting the microstructure of the material as well as the mobility of the chain segments, which in turn affects the final properties of the material.^[^ ^60^ ^]^ The ester group has greater polarity, and the interchain forces when polyester as the soft segment are usually greater than those of polyether, so the resulting polyurea material may have higher modulus and strength. It has also been found, however, that polyether‐based polyureas typically exhibit better microphase separation due to lower interactions between the soft and hard chain segments, leading to superior overall performance.^[^ ^75^ ^]^ PCL, PTMEG, and PC with crystallizable segments could be applied as the preferred precursor of robust self‐healing polyurea synthesis.^[^ ^59^, ^76^, ^77^, ^78^ ^]^ The superior performance is largely attributed to the fact that these soft segments possess suitable effective length to provide a lower crystallization energy barrier during the stretching process, which is capable of generating a hysteretic strain‐induced crystallization (SIC) phenomenon, allowing the material to achieve self‐reinforcement undergoing large deformations.^[^ ^54^, ^76^ ^]^ As the process is reversible, it allows the material to maintain its viscoelasticity in its original or retracted state. The molecular weight of the soft segments significantly affects the polyurea microphase separation and the degree of ordering/crystallization within the hard domains.^[^ ^79^, ^80^, ^81^, ^82^, ^83^, ^84^ ^]^ In the presence of just low molecular weight amines, the hard domains may change from a dispersed phase to a matrix phase, and it is only when the molecular weight of the soft segments exceeds a specific threshold that dispersed hard domains are formed, with a consequent increase in material mechanical properties.^[^ ^81^, ^85^ ^]^ The molecular weight of the soft segments should also not be too high (generally less than 5000 g·mol^−1^) to avoid excessive physical entanglement, which is detrimental to the self‐healing function.^[^ ^48^, ^85^, ^86^ ^]^

### 2.3 Chain Extender

In the absence of chain extenders, the urea bonds in polyurea are relatively distant from each other. Chain extenders are typically amine‐terminated low molecular weight molecules (with a functionality ≥ 2) that react with isocyanates and their prepolymers to increase the volume of the hard segments, thereby improving the final properties of the polyurea.^[^ ^87^, ^88^, ^89^, ^90^, ^91^ ^]^ The choice of different chain extenders allows for the customization of different hard domain structures, leading to distinct properties of the polyurea.^[^ ^87^ ^]^ Aliphatic chain extenders exhibit higher reactivity than aromatic chain extenders.^[^ ^15^ ^]^ Aromatic chain extenders result in higher deformation energy than aliphatic chain extenders. It has been shown that by utilizing the strengthening effect induced by aromatic chain extenders, the energy dissipation capacity of the material is improved and structural damage is limited.^[^ ^90^ ^]^ Chemically crosslinked points can be introduced in polyurea by using chain extenders containing branched structures, thus improving the polyurea performance through proper chemical cross‐linking.^[^ ^80^, ^91^ ^]^ More importantly, by introducing reversible chemical reactions (e.g., disulfide bonds,^[^ ^63^, ^92^ ^]^ hindered urea bonds ^[^ ^93^, ^94^ ^]^) into the diamine structure, the chain extender can impart dynamic properties to polyurea, and these reversible interactions are central to the concept of dynamic chemistry.^[^ ^95^ ^]^ Therefore, the choice of chain extenders is crucial, not only determining the ability of the material to recover from damage to some extent, but also allowing precise tuning of mechanical properties such as strength and toughness by controlling the type of dynamic interactions.

### 2.4 The Proportion of Each Component

Theoretically, the stoichiometric ratio of the ─NCO group to ─NH_2_ and ─OH group (i.e., the isocyanate index) should normally remain equimolar. In actual experiments and production processes, the isocyanate component is often controlled in slightly excessive amounts. This can be explained by the fact that the high reactivity of isocyanates means that they are prone to side‐react with water to generate urea bonds, and a slight excess of isocyanate components not only compensates for the losses caused by the side‐reaction, but also improves the cross‐linking density of the system, which is conducive to the enhancement of material mechanical properties.^[^ ^96^ ^]^ However, if the isocyanate index is greater than 1.1, it will exacerbate the occurrence of side reactions, which is not only a waste of raw materials, but also eventually likely to cause the material foaming and performance degradation.^[^ ^96^, ^97^ ^]^ Another important parameter in the regulation of polyurea formulations is the hard segment content, which refers to the mass fraction of isocyanate and chain extender in the system.^[^ ^98^ ^]^ Typically, as the hard segment content increases, the average length of the hard segment and the degree of microphase separation of the material increase, and the strength and modulus of the material increase due to the hard phases acting as physical cross‐links and filler reinforcement, while the stretchability of the material decreases.^[^ ^54^, ^98^, ^99^ ^]^ When the hard segment content reaches a specific critical value, continuing to increase the hard section content will cause a phase inversion, which means that the hard phase gradually connected, from the original dispersed phase into a continuous phase, meanwhile, the filler enhancement effect of the hard phase is no longer obvious and the strength of the material decreases.^[^ ^54^, ^89^, ^100^, ^101^, ^102^, ^103^ ^]^ The hard domain is an important limiting factor in the healing process, and typically, the temperature required for healing gradually increases as the hard segment content increases and the healing process becomes more difficult.^[^ ^40^ ^]^ According to existing studies, the hard segment content in polyureas typically ranges between 20% and 50% to achieve a balance between mechanical properties and self‐healing capabilities.

## 3 Design Strategies for Mechanically Robust Self‐Healing Polyurea

In the field of self‐healing polyureas, the various dynamic chemical reactions including dynamic covalent and non‐covalent interactions, have been summarized in detail and comprehensively in a number of papers.^[^ ^48^, ^49^, ^51^ ^]^ In our observation, the key to obtaining high‐performance self‐healing polyurea materials lies not only in the mere selection of dynamic bonds, but more in the search for suitable strategies and novel mechanisms to circumvent the contradictions of individual properties, which require rational design of the polymer chain structure from the perspective of chemistry as well as precise regulation of the microphase structure. In this section, we focus on the research progress of various types of mechanically robust self‐healing polyurea materials in recent years, as well as analyzing and summarizing their synthetic strategies from a chemical point of view.

### 3.1 Non‐Covalent Cross‐Linking and Microphase Control Strategies

In contrast to covalent bonding, non‐covalent crosslinking can lead to increased mechanical properties of materials without sacrificing the ductility and toughness through noncovalent interactions, such as hydrogen bonding and metal coordination interactions, which offer advantages over covalent bonds.^[^ ^104^, ^105^ ^]^ H‐bonding is the most prevalent noncovalent interactions in designing high‐performance polyurea materials, which features directionality, reversibility, and versatility.^[^ ^29^, ^106^ ^]^ On one hand, H‐bonding interactions are widely recognized between urea and urethane bonds. Introducing appropriate H‐bonding interactions can effectively enhance the strength and toughness of polyurea, as the physical cross‐linking network between the chain segments of the polyurea is formed by H‐bonds, which strengthens the material and dissipates the energy efficiently under the influence of external forces. On the other hand, the dynamic characteristics of hydrogen bonds inherently offer the possibility of realizing self‐healing.^[^ ^51^ ^]^ The degree of hydrogen bonding within the polymer network is closely related to the state of the hard domains. Many researchers have optimized the microphase structure by regulating the hydrogen bonds in polyurea to achieve a balance between various properties.

#### 3.1.1 Construction of Multilevel Non‐Covalent Cross‐Linking

**Figure 3:**
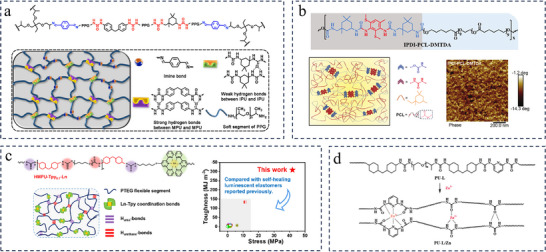
a) Synthetic route and the structural schematic of polyurea network which contains both dynamic imine bonds and hierarchical hydrogen bonds. Reproduced with permission.^[^ ^108^ ^]^ Copyright 2020. American Chemical Society. b) Schematic structural diagram and AFM phase diagram of the IPDI‐PCL‐DMTDA sample. Reproduced with permission.^[^ ^107^ ^]^ Copyright 2024. Wiley. c) Chemical structure illustration of HMPU‐Tpyn‐Ln. Comparison of the toughness and stress of HMPU‐Tpy0.1‐Ln with these of the reported self‐healing luminescent polymers. Reproduced with permission.^[^ ^109^ ^]^ Copyright 2023. American Chemical Society. (d) Scheme of the synthetic procedure of the PU‐L and PU‐L/Zn. Reproduced with permission.^[^ ^110^ ^]^ Copyright 2023, American Chemical Society.

Weak hydrogen bonding crosslinking can lead to degradation of the polyurea material properties, while excessively strong hydrogen bonding interactions tend to cause agglomeration of the polyurea, resulting in stress concentration and reduced energy dissipation efficiency.^[^ ^107^ ^]^ Thus, optimizing the strength and density of H‐bonds is seen as a viable approach to further improve the mechanical properties of polyureas. One common strategy is to build multilevel hydrogen bonds of varying strengths and/or densities in one polyurea network, which can be achieved by modulating isocyanates and/or soft segments and/or chain extenders. Taking Zhang's work as an example, by adjusting the mass ratio of the soft segments with different chain length (H‐bond density) and the types of isocyanates (H‐bond strength), they reported a water‐enabled room‐temperature self‐healing polyurea with super‐strong strength (41.2 MPa), large stretchability (823%), and excellent toughness (127.2 MJ·m^−3^) (**Figure** **3**a).^[^ ^108^ ^]^ The increase in the mass ratio of PPG (Mn ≈ 400)/PPG (Mn ≈ 2000) can effectively improve the density of H‐bonds within material, contributing to the significant increase in the tensile strength. The molar ratio of MDI/IPDI was adjusted to control the relative strength of the H‐bonds within polyurea, where strong quadruple H‐bonds formed between 4,4′‐methylenebis(phenylurea) (denoted as MPU) and MPU and relatively weak single or dual H‐bonds formed between isophorone bisurea (denoted as IPU) and IPU. The synergistic effect of the multiple dynamic interactions including hierarchical H‐bonds and dynamic imine bonds within polyurea networks endowed it with water‐enabled self‐healing ability with a high healing efficiency of 92.2% (healing for 72 h at room temperature). The polyurea elastomers with diverse hard microdomains containing multilevel hydrogen bonds were constructed utilizing different isocyanates and chain extenders by Dai and co‐workers.^[^ ^107^ ^]^ Among them, the polyurea elastomer synthesized from IPDI, PCL, and 2,4‐diamino‐3,5‐dimethylthiotoluene (DMTDA) exhibited excellent mechanical properties, including a tensile strength of 35.2 MPa, a toughness of 165.5 MJ·m^−3^ and a fracture energy of 125.9 MJ·m^−2^ (Figure 3b). The presence of smaller, densely, and uniformly distributed hard microdomains constructed by hierarchical H‐bonding interactions in the polymer network is a critical step in balancing mechanical strength and toughness, favoring efficient energy dissipation upon the external forces. In addition, due to the dynamic nature of H‐bonding interactions, elastomers with incision could be easily healed at 100 °C for 36 h with a healing efficiency of ≈100% and recycled under heating and pressure conditions.

H‐bonds have been used to combine with coordination bonds in one polyurea to further optimize the mechanical strength, toughness, and self‐healing ability by constructing robust multilevel non‐covalent crosslinking networks.^[^ ^36^ ^]^ Li and coworkers designed a luminescent and ultrastrong self‐healing elastomer through the strategy of integrating multiple hydrogen bonds and polydentate chelating lanthanide‐terpyridine (Ln^3+^‐Tpy) coordination interactions in one network (Figure 3c), based on PTMEG, HMDI, and Ln^3+^‐Tpy coordination components.^[^ ^109^ ^]^ The synergic effect of dense Ln^3+^‐Tpy coordination bonds and multiple H‐bonds is believed to be responsible for the significantly improved mechanical strength (40.9 MPa) and toughness (308.63 MJ·m^−3^) of HMPU‐Tpy_0.1_‐Ln samples. The dynamic nature of Ln^3+^‐Tpy coordination bonds and hydrogen bonds allows for the repair of material damage under thermal stimulation (healing efficiency of 63.9% after self‐healing at 100 °C for 24 h). Based on the same strategy, our team also reported the synthesis of a robust and self‐healing polyurea material, which contains a dual metal coordination structure of Zn(II)‐urea and Zn(II)‐diamidepyridine as well as dense hydrogen bonds (Figure 3d).^[^ ^110^ ^]^ Due to the synergistic enhancement of dynamic coordination bonds and hydrogen bonds in the supramolecular polyurea network, the obtained PU‐L/Zn‐3 possesses a combination of excellent mechanical properties (toughness of ≈222.68 MJ·m^−3^) and high‐efficiency self‐healing properties with healing efficiency of 95.7% after treatment at 50 °C for 4 h.

#### 3.1.2 Hard Domain Dynamic Reinforcement Strategy

**Figure 4:**
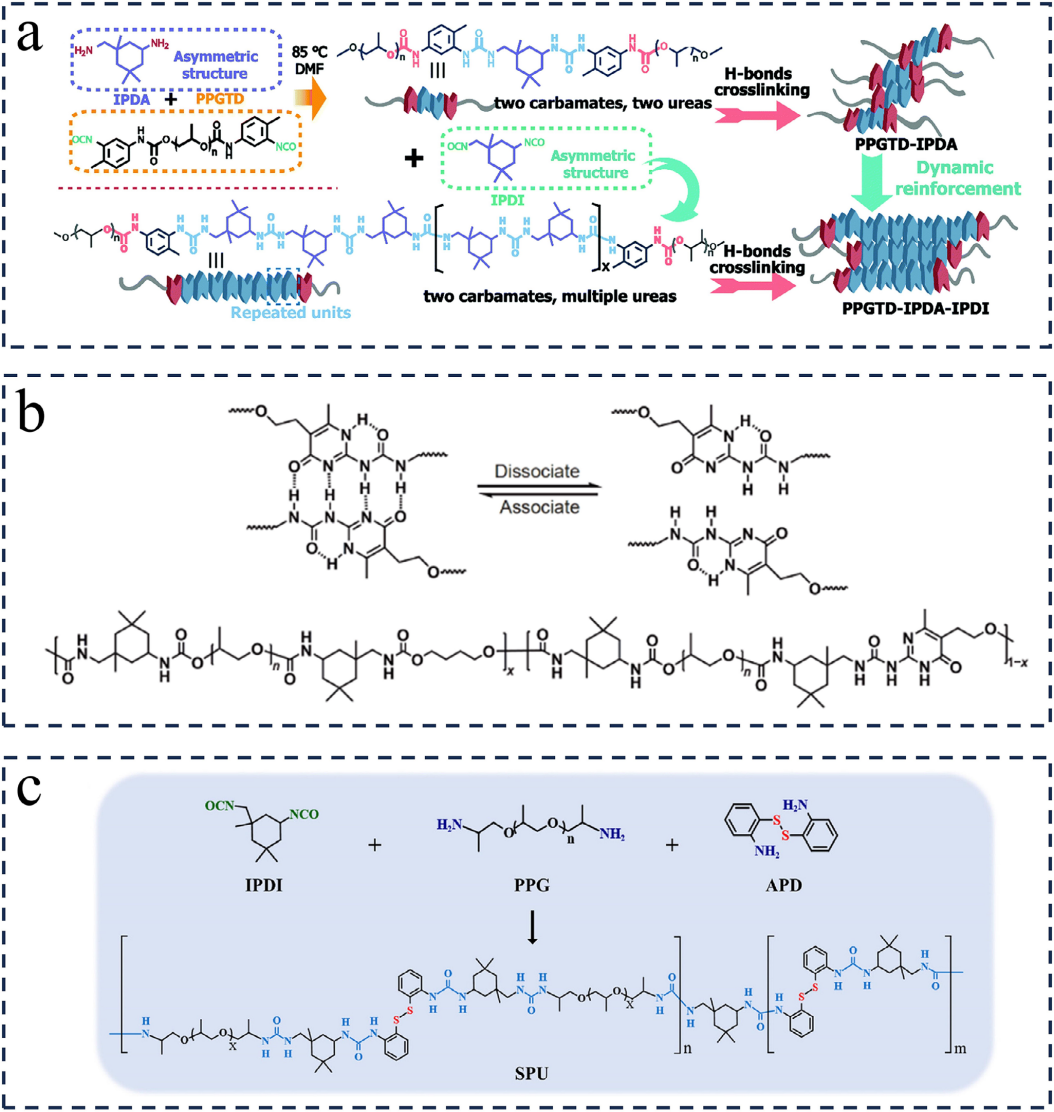
a) Synthetic routes and the schematic chemical structures of PPGTD‐IPDA and PPGTD‐IPDA‐IPDI elastomers. Reproduced with permission.^[^ ^39^ ^]^ Copyright 2021, Royal Society of Chemistry. b) Schematic diagram of association and dissociation of the UPy dimer. The structure of PPG‐mUPy. Reproduced with permission.^[^ ^113^ ^]^ Copyright 2019, Springer Nature. c) Synthesis route and chemical structure of the SPU samples. Reproduced with permission.^[^ ^95^ ^]^ Copyright 2022, Wiley.

To regulate the strength and distribution of hydrogen bonds, a novel strategy of dynamic reinforcement for hard domains was proposed by Fu and co‐workers, the key idea of which is to stiffen and strengthen the hard domains at the molecular level while keeping dynamic adaptiveness and responsiveness.^[^ ^39^ ^]^ Specifically, as shown in **Figure** **4**a, substantial urea bonds were introduced into the polymer chain by constructing repetitive “‐IPDI‐IPDA‐” units within the hard domains, which transformed the loosely stacked hierarchical urethane/urea hydrogen‐bonded hard domains into urea‐dominated hydrogen‐bonded hard domains. The substantially increased number of sacrificial H‐bonds within the IPDI‐modified hard domains not only caused a significant increase in mechanical strength (up to 33.4 MPa), but also provided an effective means of energy dissipation, imparting networks with pronounced toughness (up to 503.3 MJ·m^−3^). Due to the enormous asymmetric alicyclic rings that effectively prevented crystallization of the hard domains as well as the rapid and reversible association/dissociation of IPDI‐modified hard domains, polyurea elastomers (PPGTD_0.4_‐IPDA_1.0_‐IPDI_0.6_ as an example) exhibited a healing efficiency of 93% with the assistance of trace solvent. Controlling the IPDI content was effective in tailoring the microphase's structure and morphology, thereby switching the mechanical properties of elastomers on demand. Similarly, Guo et al. relied on multiple repeating asymmetric aliphatic rings of hard segments (formed by IPDI and IPDA) and soft segments with strong binding capacity (PTMEG) to form excellent polyurea materials with tensile strengths of up to 45 MPa, elongation at break of 850%, tear strength of up to 118.776 KJ·m^−3^ and its mechanical properties almost completely restored after healing at 80 °C for 12 h.^[^ ^111^ ^]^

#### 3.1.3 “Phase‐Locked” Strategy

The “phase‐locked” strategy, originally proposed by Wang et al. in 2018, refers to the locking of dynamic bonds belonging to the weak part of the chain in the hard microphase domain by means of the microphase separation of the thermoplastic polyurethane (TPU) for balancing the mechanical robustness and self‐healing efficiency of TPU elastomers.^[^ ^41^ ^]^ The strategy is equally valid for polyurea, and is comparable to introducing a thermal‐triggered switch inside the material.^[^ ^112^ ^]^ Upon external mechanical forces, the dynamic bonds are locked and cannot be exchanged below the *T_g_* /*T_m_* of hard domain, allowing for excellent mechanical properties. The locked bonds can be easily activated to be highly dynamic above the *T_g_* /*T_m_*, endowing the materials with effective self‐healing capabilities. Wang et al. developed polyurea‐urethane materials containing ureidopyrimidone (UPy) motifs.^[^ ^113^ ^]^ The dense quadruple H‐bonds between the UPy motif induce microphase separation and hard domain aggregation into microcrystals, which are stable at room temperature, ensuring the high mechanical strength and toughness of the materials (Figure 4b). When thermal stimulation is applied to the system (higher than *T_m_* of the hard domain microcrystal), dynamic H‐bonds is activated, endowing the material with good self‐healing property. One representative sample achieved a good balance of mechanical properties (tensile strength of 20.62 MPa and toughness of 100.49 MJ·m^−3^) and self‐healing efficiency (93% at 80 °C within 24 h). Our team employed this strategy to synthesize a high‐performance self‐healing polyurea (SPU).^[^ ^95^ ^]^ Specifically, PPG was used as the soft segment, IPDI, and a dynamic disulfide bond‐containing chain extender, 2‐Aminophenyl disulfide were used as the hard segments to synthesize the polyurea (Figure 4c). This structural design leads to irregular stacking of the molecular chains and the strong hydrogen bonding in the hard segments, thus preventing crystallization effectively and resulting in strong microphase separation. The optimized sample exhibited high mechanical strength (tensile stress of 21.2 MPa), high rigidity (Young's modulus of 248.1 MPa), excellent energy absorption (toughness of 139.7 MJ·m^−3^), and good self‐healing property (self‐healing efficiency of 92.7% after 48 h of treatment at 120 °C). The phase‐locked strategy provides a valuable reference for the design of high‐performance self‐healing polyurea materials.

#### 3.1.4 Strain‐Induced Crystallization

Most high‐performance self‐healing polyureas experience strain‐induced crystallization (SIC) during stretching. The SIC domains act as physical crosslinking junctions and reinforcing phase, resulting in remarkable strengthening and toughening.^[^ ^44^, ^45^, ^114^ ^]^ The realization of SIC not only requires the modulation of the type of soft segments, i.e., the selection of crystallizable soft segments such as PTMEG, PCL, PC, etc. with suitable effective lengths, which provide lower crystallization energy barriers during the stretching, but also is closely related to the structure of the hard domains and internal H‐bonds. The hierarchical interactions as sacrificial and dynamic bonds within these elaborately designed hard domains not only dissipate stress energy and produce self‐enhancement effect based on SIC, but also provide effective dynamic bonds exchange upon damage.^[^ ^42^, ^43^, ^78^ ^]^ The number and binding energy of hydrogen bonds, the aggregation state of the hard segments, and the size of the hard domains significantly affect the dissociation and association of H‐bonds within the hard domains, and thus the production of SIC.

**Figure 5:**
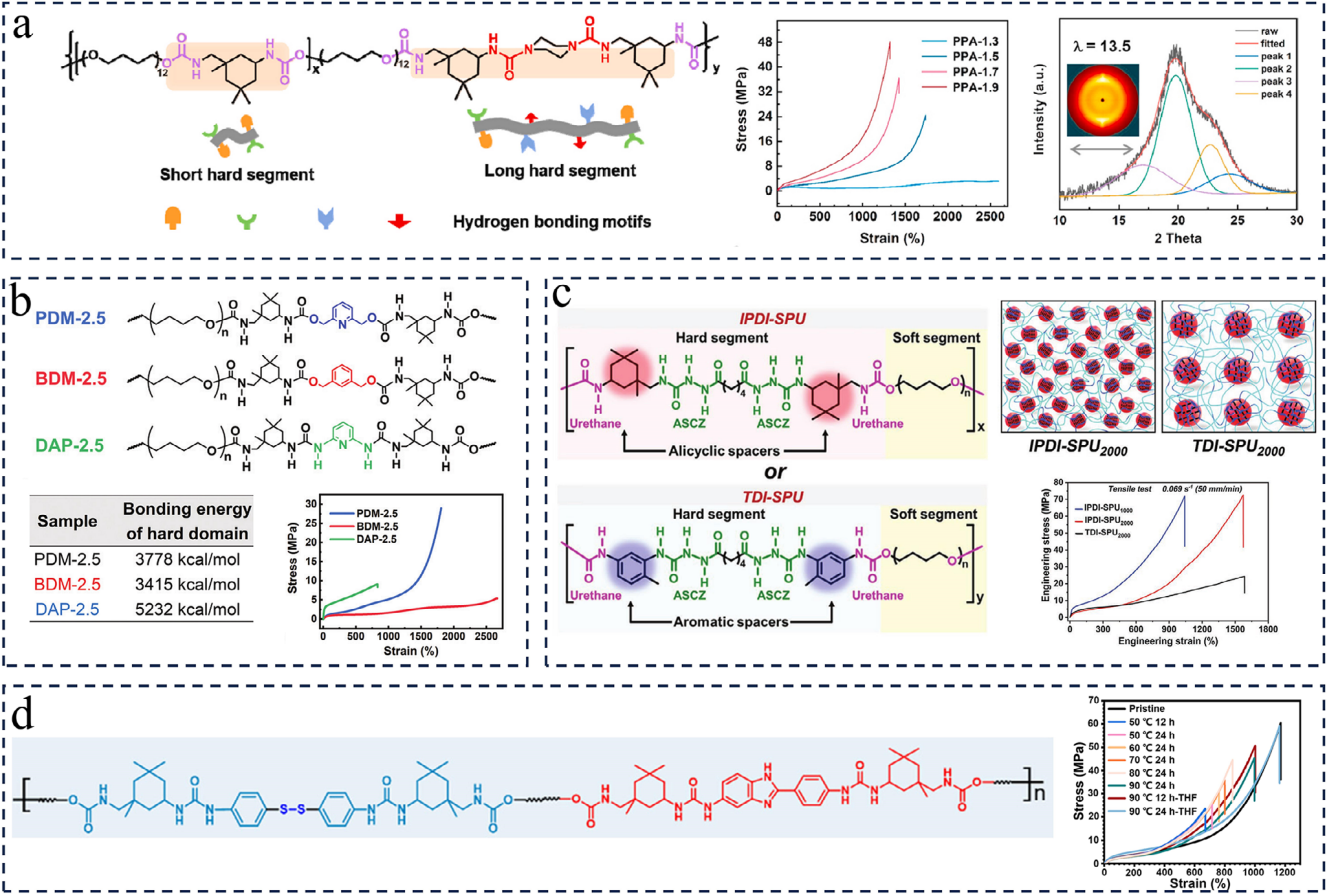
a) Molecular structure of PPA‐based elastomers embedded with short and long hard segments. Stress‐strain curves of PPA‐based elastomers. One‐dimensional WAXD raw and fitted curves of stretched PPA‐1.7 elastomer, and inset shows the two‐dimensional WAXD pattern. Reproduced with permission.^[^ ^42^ ^]^ Copyright 2021, Elsevier. b) Schematic structure, binding energies of the hard domains, and typical stress‐strain curves of PDM‐2.5, BDM‐2.5, and DAP‐2.5. Reproduced with permission.^[^ ^78^ ^]^ Copyright 2021, Royal Society of Chemistry. c) Synthetic routes, schematic illustration of the microstructures, and engineering stress‐strain curves of the IPDI‐SPU and TDI‐SPU elastomers. Reproduced with permission.^[^ ^32^ ^]^ Copyright 2021, Wiley. d) Schematic diagram of the preparation procedure of polyurea elastomer and the stress‐strain curves for the healed PU‐5/5 after different healing times at different temperatures. Reproduced with permission.^[^ ^115^ ^]^ Copyright 2024, Royal Society of Chemistry.

It has been reported that the hydrogen bonding behavior of the polyurea network was modulated by creating weak short hard segments and strong long hard segments and adjusting their relative contents to ensure the SIC of soft chain segments, and ultimately PPA‐1.7 elastomers were obtained with excellent comprehensive performances in tensile strength (38.5±1.3 MPa), toughness (134.6±7.0 MJ·m^−3^), puncture resistance (697.9 mJ), elasticity and room temperature self‐healability (**Figure** **5**a).^[^ ^42^ ^]^ Short hard segment unit contain merely two urethane motifs bonded to IPDI moiety, which perform weak hydrogen bonding due to high steric hindrance of bulky IPDI moiety. They are loosely and irregularly assembled resulting in a low binding energy. While the long hard segment unit contains two more urea motifs bonded to PPA moiety. Owing to the symmetry ring of PPA and the high cohesive energy density of urea, the urea‐urea hydrogen bonds are more strongly bonded, whereby the long hard segments are tightly assembled in a relatively regular manner and play a key role in generating SIC.^[^ ^67^ ^]^ AFM and SAXS analyses show that PPA‐1.7 features loosely stacked hard domains, which are uniformly embedded in the soft phase, leading to a suitable microphase separation, resulting in a balance between mechanical properties and viscoelastic behavior.

Fu and coworkers regulated the phase by rationally selecting asymmetric and bulky hard segments as well as optimizing the content of hard segments, as shown in Figure 5b.^[^ ^78^ ^]^ The resulting loosely stacked hard domains are not only conducive to the dissipation of stress energy during stretching, thus generating SIC of PTMEG chains during large deformation for the purpose of strengthening and toughening, but also facilitate the chain mobility for heat‐responsive dynamic exchange of hydrogen bonds and oxime‐carbamate, enabling self‐healing of damage. Precise modulation of H‐bonds within the hard domains and the structure of the hard domains can be achieved by changing the structure of the chain extender and thereby utilizing SIC for strengthening and toughening.^[^ ^43^ ^]^ For example, for BDM‐2.5, the carbamate‐carbamate H‐bonds within the hard domains are too weak to maintain the oriented soft segments. Therefore, both the hard and soft segments continuously perform mutual displacement and, as a result, the PTMEG chains are unable to align into SIC domains in sufficient amounts and in large sizes. For DAP‐2.5, the urea‐urea H‐bonds in hard domains are too strong to efficiently dissipate the stress‐energy during stretching. The soft phase fails to withstand the external stress and breaks before the PTMEG chains are completely disentangled or oriented in alignment. Compared to this, PDM‐2.5 benefits from loosely stacked but hierarchically associated H‐bonds and exhibits a pronounced SIC phenomenon during stretching. The carbamate‐carbamate H‐bonds with relatively low binding energy facilitate dissipating stress energy during stretching; with the increase of strain, the PTMEG chains gradually align along the stretching direction, and resulting in the crystalline domain; meanwhile, the stronger pyridine‐carbamate H‐bonds delay their mutual dissociation and maintains the SIC domains. Once the pyridine‐carbamate H‐bonds fail to sustain the increased external force, they dissociate and the material breaks, ultimately yielding sufficient mechanical strength (29.0 ± 0.9 MPa) and toughness (121.8 ± 8.5 MJ·m^−3^) to be quite prominent in room‐temperature self‐healing polyurea/polyurethane materials.

The density and binding energy of hydrogen bonds in hard segments were modulated by Liu et al. by changing the type of isocyanate.^[^ ^32^ ^]^ As illustrated in Figure 5c, the flexible alicyclic hexatomic spacers in the IPDI‐SPU_2000_ facilitate the formation of higher density of smaller hard domains (i.e., denser H‐bond arrays), and the flexible PTMEG chains are more firmly interlocked through high‐density H‐bonds, which can not only dissipate energy through dynamic breakage and reformation of H‐bonds under external forces, but also generate the SIC effect to realize the self‐enhancement of the material. Whereas the high rigidity of the aromatic spacers in the TDI‐SPU_2000_ polymer inhibits the conformational compliance of hard segment, leading to lower‐efficiency H‐bonds and larger‐size hard domains, resulting in relatively low mechanical properties. Wu et al. proposed an innovative design strategy that combined symmetric/asymmetric chain extenders to create large yet disordered hard domains within polyurea elastomers (Figure 5d).^[^ ^115^ ^]^ Importantly, this microphase structural feature leads to a small free‐volume fraction, prominent SIC, and energy dissipation, resulting in polyurea elastomer exhibiting excellent mechanical strength (60.7 MPa) and toughness (177.9 MJ m^−3^). As well, the loose stacking of disordered hard domains imparts the network with high relaxation dynamics and low cohesive energy, leading to a healing efficiency of 97.8% after healing at 90 °C for 24 h with the assistance of tetrahydrofuran.

In summary, the construction of hard domains that are disordered/loosely stacked and hierarchically correlated intra‐hard‐domain hydrogen bonds is the key to self‐enhancement using the SIC strategy. The loose stacking of the hard segments facilitates the dissipation of strain energy, while the strong interactions are responsible for maintaining the directional alignment of the soft segments, resulting in a self‐reinforcing that greatly increases the strength and toughness of the material.

### 3.2 Dynamic Covalent Cross‐Linking

**Figure 6:**
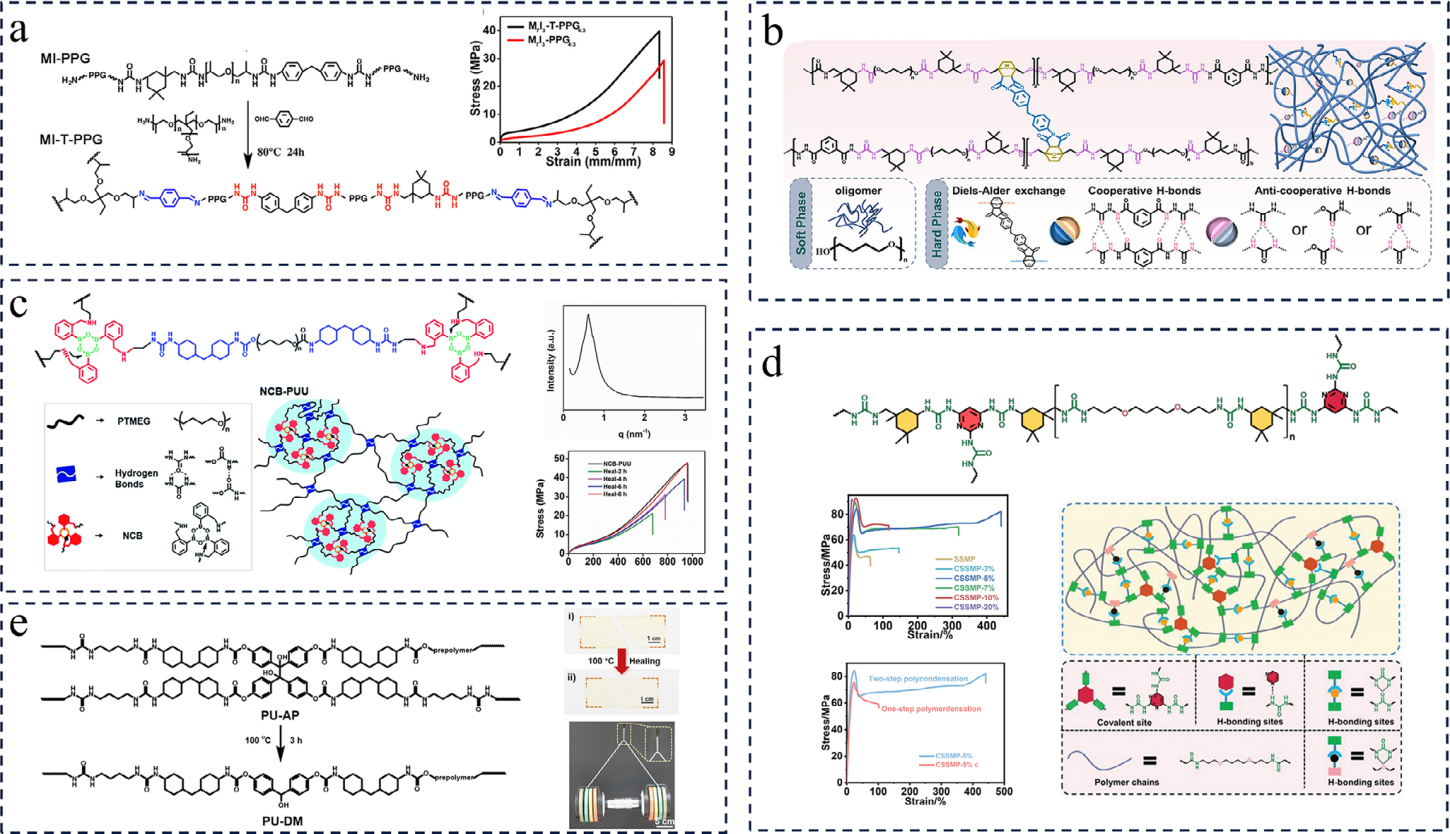
a) Synthetic route and stress‐strain curves of M7I3‐T‐PPG4:3 and M7I3‐PPG4:3. Reproduced with permission.^[^ ^108^ ^]^ Copyright 2020, American Chemical Society. b) Three‐dimensional network conformation of macromolecules with double cross‐linked structure. Reproduced with permission.^[^ ^117^ ^]^ Copyright 2023, Elsevier. c) Schematic structure and SAXS curve of NCB‐PUUs. Stress–strain curves of the NCB‐PUU sheet healed with the assistance of the water/ethanol mixture (volume ratio 2:1). Reproduced with permission.^[^ ^120^ ^]^ Copyright 2021, Royal Society of Chemistry. d) Schematic illustration of the chemical structure and various constituent groups of the CSSMP network. The stress–strain curves. Reproduced with permission.^[^ ^121^ ^]^ Copyright 2024 Wiley. e) Chemical structure of PU‐DM derived from cleaved PU‐AP. Schematic of PU‐DM healing and lifting a heavy object. Reproduced with permission.^[^ ^65^ ^]^ Copyright 2024, Wiley.

The chemical cross‐linking can prevent slippage between polyurea molecular chains, which plays a crucial role for mechanical strength and stiffness, but usually at the expense of extensibility and dynamic properties.^[^ ^116^ ^]^ In order to break the aforementioned contradiction, researchers have proposed the design concept of constructing dynamic covalent crosslinked networks, in which the typical strategy is that the crosslinking agents in the polyurea directly connect with dynamic bonds to increase the dynamics of the chemical crosslinked network. For instance, the diamino‐terminated linear polyurea chains were further crosslinked by dynamic imine bonds based on the Schiff reaction by Zhang et al. to prepare cross‐linked polyureas (**Figure** **6**a).^[^ ^108^ ^]^ Their results showed that the mechanical properties of the M_7_I_3_‐T‐PPG_4:3_ samples (with imine‐bond cross‐linking) were significantly improved and the elongation at break only slightly decreased as compared to the M_7_I_3_‐PPG_4:3_ (without imine‐bond cross‐linking). Dynamic interactions can also act directly as cross‐linkers to introduce chemical cross‐links in the polyurea network. Zhao et al. prepared a double‐crosslinked polyurea network by introducing the dynamic covalent crosslinking of Diels‐Alder bonds and physical crosslinking of high‐density hydrogen bonds, which exhibits high strength (72.5 MPa), toughness (235.7 MJ·m^−3^) and a self‐healing efficiency of 86% under thermal stimulus.^[^ ^117^ ^]^ As shown in Figure 6b, bismaleimide acts as cross‐linking agent and undergoes an addition reaction with 2,5‐furandimethano, thus introducing dynamic covalent bonds of D‐A bonds in polyurea to form a three‐dimensional cross‐linking network. Dynamic covalent cross‐linking of D‐A bonds could maintain the relative positions of polymer chains during stretching and efficiently maintain the stability of the three‐dimensional cross‐linked network. Simultaneously, the physical crosslinking of high‐density H‐bonds contributes to the toughness by dissipating strain energy. The combination of both crosslink types leads to remarkable toughness and strength. Moreover, the dynamic reversibility of H‐bonds and D‐A bonds at high temperatures endows the polyurea with repairable properties.

Nitrogen‐coordinated boroxines (NCBs), featuring not only a tripodal molecular architecture that allows for high cross‐linking density of the polymer network but also higher reversibility at room temperature, are good candidates for the manufacture of high‐strength and self‐healable polyurea.^[^ ^118^, ^119^ ^]^ Based on the rational design of the phase separation structure, a room‐temperature healable and mechanically robust polyurea‐urethane was fabricated by cross‐linking the linear prepolymers of PTMEG and HMDI with NCBs (Figure 6c).^[^ ^120^ ^]^ The microphase separation with a periodicity of ≈10.3 nm was calculated by SAXS curve. The high density of dynamic cross‐linking of NCBs and H‐bonds as well as the appropriately scaled phase‐separated domains result in high strength (≈47 MPa) and toughness (≈190 MJ·m^−3^) of the obtained samples (NCB‐PUUs). In addition, due to the highly dynamic reversibility of the network, the NCB‐PUUs can be effectively healed and recycled at room temperature with the assistance of ethanol or a water/ethanol mixture.

Excessive or unevenly distributed chemical cross‐linking may lead to a concentration of deformation within the matrix, which exhibits macroscopic inhomogeneity of deformation and a decrease in the comprehensive mechanical properties. Theoretically, it is necessary to precisely regulate the position and density of chemical cross‐linking points on the basis of elaborate molecular engineering in order to equilibrate and improve the various properties of the material. Based on this, inspired by the structure of muscle fibers, Fu and coworkers reported an innovative approach for fabricating high‐performance polyureas by introducing precisely slight chemical cross‐linking into a hierarchical hydrogen‐bonded network of supramolecular polymers (Figure 6d).^[^ ^121^ ^]^ Specifically, these polyureas were synthesized via a one‐pot two‐step polycondensation between IPDI, diamine monomers (1,12‐Diamino‐4,9‐dioxadodecane), and triamine monomer (2, 4, 6‐triaminopyrimidine). The representative CSSMP‐5% sample demonstrates superior mechanical properties, including Young's modulus of 1347 MPa, fracture stress of 84.1 MPa, and toughness of 312.7 MJ·m^−3^. Moreover, the recycling and healing efficiency of CSSMP‐5% exceeded 95% after undergoing reprocessing five times (150 °C, 0.5 MPa, 5 min) and subsequent healing at room temperature with the assistance of isopropanol and applied pressure for 12 h. The excellent mechanical properties are attributed to the following three aspects: i) The presence of multilevel intermolecular hydrogen bonds within the polymer network, including those created by urea‐urea, urea‐pyrimidine, and urea‐ether, facilitates the energy dissipation under external forces. ii) The appropriate chemical crosslinking density of polyurea network not only preserves interchain sliding as a means of energy dissipation but also provides a significant bridging effect in load transfer. iii)The use of a two‐step method instead of a one‐step method allows for precise positioning of cross‐linking sites, ensuring uniform distribution of forces during stretching and overall integrity of the polymer network. Four‐arm aromatic pinacol (AP) cross‐linkers are designed for the synthesis of mechanically robust polyurea‐urethane thermosets (PU‐AP) in a recent work (Figure 6e), which exhibits an extremely high breaking strength of 95.5 MPa, Young's modulus of 248.7 MPa, and toughness of 473.6 MJ·m^−3^.^[^ ^65^ ^]^ Interestingly, when heated in DMAc at 100 °C, the AP units in PU‐AP be cleaved into diphenylmethanol (DM) units, resulting in the transformation of the PU‐AP thermosets into linear PU‐DM elastomers that exhibit a breaking strength of 74.2 MPa, elongation at break of 1231%, and toughness of 312.3 MJ·m^−3^. The dynamic dissociation of H‐bonds in the PU‐DM elastomer matrix and nanodomains under heating conditions enhances the mobility of the polymer chains and endows PU‐DM with self‐healing and reprocessing capacity. Synthesis of cleavable thermosets using stimulus‐cleavable crosslinkers provides a new idea for the development of self‐healing and recyclable high‐performance polyurea materials.

### 3.3 Construction of Modular Polyurea Based on Biomimetic Strategy

**Figure 7:**
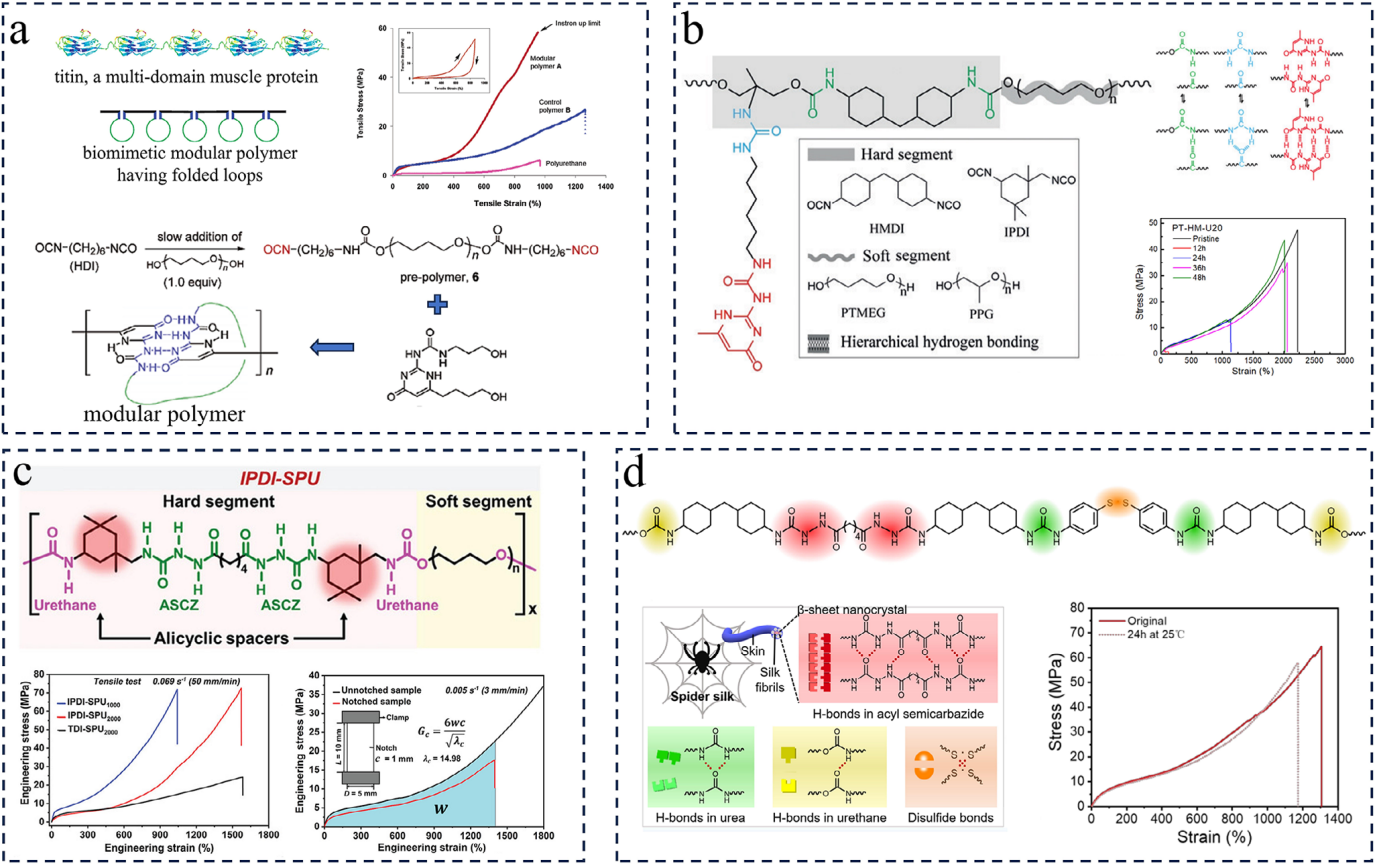
a) A small section of titin, which has 200–300 repeating immunoglobulin domains. The design of modular polymer containing multiple loops held by secondary forces. The Synthesis of the UPy‐containing polymers and stress–strain curves for the control and modular polymers. Reproduced with permission.^[^ ^123^ ^]^ Copyright 2004, American Chemical Society. b) Illustration of the self‐healing and self‐recovery process of an elastomer by the dynamic reformation of its modular structure consisting of hierarchical hydrogen‐bonding interactions. Engineering stress–strain curves of the PT‐HM‐U20 self‐healed at 80 °C for different times and at different temperatures for 48 h. Reproduced with permission.^[^ ^128^ ^]^ Copyright 2018, Wiley. c) Structure of the IPDI‐SPU_2000_ elastomers. Typical engineering stress–strain curves of the elastomers as well as the unnotched and notched IPDI‐SPU_2000_ sample. Reproduced with permission.^[^ ^32^ ^]^ Copyright 2021, Wiley. d) Molecular structure of AD/2S‐PU with multiple dynamic bonds. Dynamic bond interaction in the biomimetic AD/2S‐PU. Stress‐strain curves of the original and healed samples. Reproduced with permission.^[^ ^22^ ^]^ Copyright 2022, American Chemical Society.

It remains a challenge to design synthetic polymers with a combination of damage self‐healing ability and mechanical properties such as high strength, toughness, and elasticity. In contrast, many smart strategies have evolved in nature to achieve biopolymers that combine these properties. For example, silks, cell adhesion proteins, and connective proteins existing in tissues such as muscle, seashell, and bone exhibit a remarkable combination of high strength, toughness, and, sometimes, dynamic properties which are rarely observed in a synthetic polymer.^[^ ^122^, ^123^ ^]^ Some studies revealed that the combination of excellent properties in these natural materials derives from their unique molecular and nanoscopic structures.^[^ ^124^, ^125^ ^]^ Taking titin as an example, it is a giant protein of muscle sarcomere with 200–300 repeating modules, each composed of linearly arranged nanodomains, and each domain is bound together by secondary interactions, e.g., hydrogen‐bonding, hydrophobic, and van der Waals interactions.^[^ ^123^, ^126^, ^127^ ^]^ The tunable microphase structure and rich in hydrogen bonds of polyureas allow them to achieve self‐enhancement by mimicking this strategy. Guan et al. reported the first application of this unique molecular mechanism to polymer design, using titin as a model and a strong quadruple hydrogen bonding motif, 2‐ureido‐4‐pyrimidinone (UPy), to guide the formation of loops along the polymer chain.^[^ ^123^ ^]^ The resulting modular polyurethane material successfully combines high mechanical strength and toughness due to the sequential unfolding of the loops during stretching (**Figure** **7**a). They did not, however, assess the self‐repair function of the system. In fact, the hydrogen bonding between UPy units is dynamically reversible under certain conditions. On this basis, a titin‐mimicking modular polyurea‐urethane elastomer constructed by hierarchical (single, double, and quadruple) H‐bonds was demonstrated by Li et al.^[^ ^128^ ^]^ The hierarchical H‐bonds formed with urethane, urea, and UPy groups lead to a durable network structure that has enhanced mechanical properties such as toughness and elastic recovery and is also dynamic for rapid self‐healing (Figure 7b). Different soft and rigid segments were utilized to adjust the self‐healing and mechanical properties. It was demonstrated that the remarkable toughness and recoverability are derived from the dynamic and dense H‐bonding interactions, which lead to the successive formation of rigid phases that can dissipate energy. Both the well‐defined hierarchical supramolecular interaction, and the modular structure which is similar to that of the titin, contribute to the excellent toughness (345 MJ·m^−3^) and strength (44 MPa) of fully‐cut PT‐HM‐U20 sample after self‐healing at 80 °C for 48 h.

This biomimetic strategy, which utilizes the quadruple H‐bonds domains provided by the UPy motifs as supramolecular building blocks, is an alternative approach for obtaining dynamic and robust self‐healing polyureas.^[^ ^129^ ^]^ Nevertheless, the specific dimerization of UPy based on quadruple H‐bonds is considered to set an upper limit on the reinforcement and toughening of the material. Several recent studies have shown that nonspecific supramolecular building blocks are capable of forming multimers that can further reinforce and toughen materials, representative of which is acylsemicarbazide (ASCZ) building block.^[^ ^22^, ^31^, ^32^, ^130^ ^]^ Also, spider silks with unique multi‐scale microstructures are regarded as one of the toughest materials in the world, with tiny fibers containing β‐sheet crystals and randomly coiled amorphous phases.^[^ ^19^, ^122^, ^131^ ^]^ The β‐sheet nanocrystals geometrically confined within a few nanometers and containing unique arrays of hydrogen bonds are uniformly embedded in the flexible amorphous matrix, creating unique microphase separation which should predominantly account for the excellent mechanical properties of spider silk. When subjected to external forces, the densely arranged sacrificial H‐bonds in the β‐sheet nanocrystals rupture and disperse the forces, resulting in excellent mechanical strength and toughness of spider silk. Inspired by the above two concepts, Sun et al. performed meticulous molecular engineering, and adipic dihydrazide (AD) was used as a chain extender along with IPDI and PTMEG to synthesize a polyurea‐urethane elastomer (IPDI‐SPU_2000_), whose hard segments consisted of multiple ASCZ and urethane moieties (Figure 7c).^[^ ^32^ ^]^ Such a design facilitates the formation of higher density of distinctive hard domains that exhibit smaller sizes but involve denser H‐bonds, while the flexible PTMEG chains are crosslinked by the hard domains comprised of H‐bond arrays allowing stronger interlocking of polymer chains, which is similar to the secondary structure of spider silk.^[^ ^19^, ^122^, ^131^ ^]^ In addition, the denser H‐bonds in hard domains can dissipate energy more effectively through the dynamic rupture and reformation of the H‐bonds. Thus, IPDI‐SPU_2000_ elastomers achieve an extraordinary combination of ultra‐high strength (ultimate engineering stress of ≈75.6 MPa), enormous toughness (≈390.2 MJ·m^−3^), superb crack tolerance (fracture energy of ≈215.2 kJ·m^−2^) and high elastic restorability. Furthermore, the IPDI‐SPU_2000_ elastomers are healable after damage benefiting from dynamic nature of the H‐bonds (healing efficiency ≈100% after healing at 100 °C for 36 h). Wang and colleagues introduced dynamic ASCZ moieties and disulfide bonds into the polymer backbone to design a robust room‐temperature self‐healing polyurea‐urethane (AD/2S‐PU), the structure of which is shown in Figure 7d.^[^ ^22^ ^]^ The significantly enhanced mechanical strength (64.6 MPa) of AD/2S‐PU is mainly attributed to the suitable microphase separation and the strong sextuple H‐bonding provided by the acyl semicarbazide motif, which results in a more stable polymer network. The dynamic fracture and reconstruction of H‐bonds in ASCZ, urea, and urethane motifs as well as the dynamic decomposition of disulfide bonds facilitates energy dissipation, which not only leads to high toughness (345.8 MJ·m^−3^) but also enables the AD/2S‐PU to self‐heal at room temperature after damage.

**Figure 8:**
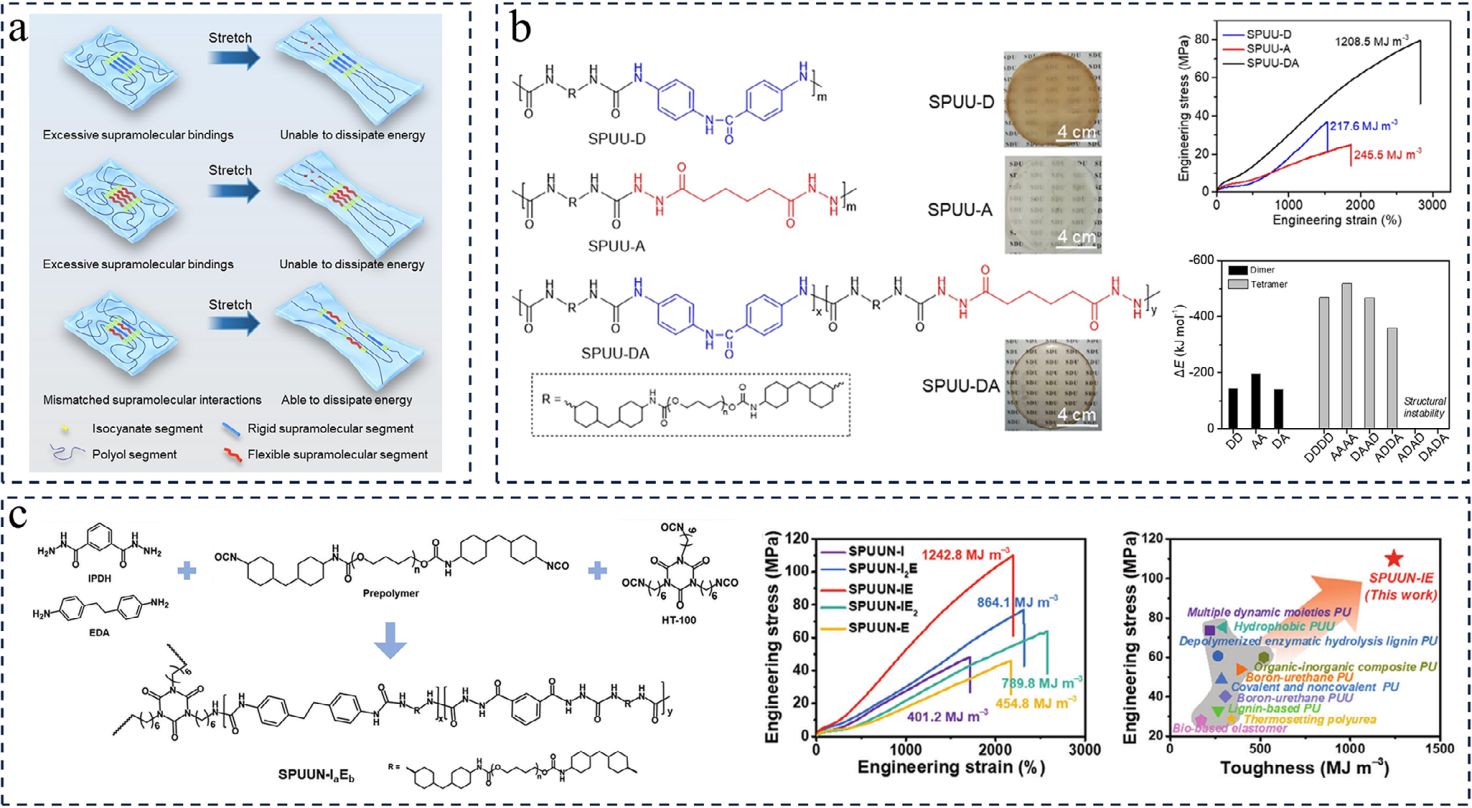
a) Toughening of elastomers by the interplay between rigid and flexible supramolecular segments. Reproduced with permission.^[^ ^133^ ^]^ Copyright 2023, Wiley. b) Synthetic routes of elastomers with rigid and/or flexible supramolecular segments. Typical engineering stress–strain curves. The binding energies of dimers and tetramers comprising rigid and/or flexible supramolecular segments. Reproduced with permission.^[^ ^133^ ^]^ Copyright 2023, Wiley. c) Schematic of the synthetic procedure employed to obtain the desired SPUUN‐I_a_E_b_ species. Typical engineering stress–strain curves for the SPUUN‐I_a_E_b_ materials. Ashby plot of tensile toughness versus the engineering stress for the thermoset elastomers with tensile toughness values >150 MJ·m^−3^. Reproduced with permission.^[^ ^134^ ^]^ Copyright 2024, Wiley.

On the basis of the above studies, the researchers found that unconstrained non‐specific multivalent binding has the potential to lead to excessive supramolecular stacking, which means stronger intrinsic interactions and strength, but not higher toughness.^[^ ^132^ ^]^ Excessive binding strength can prevent aggregates from effectively dissociating to dissipate energy under external forces. This requires more precise regulation of nonspecific binding for the purpose of balancing supramolecular binding strength and energy dissipation in the polymer network, thus further enabling toughening without sacrificing mechanical properties such as strength, elasticity, and self‐healing capability. On this basis, Wang's team proposed a novel method to toughen and strengthen thermo‐plastic polyurea materials by utilizing the mechanistic interplay between rigid and flexible supramolecular segments (**Figure** **8**a‐c).^[^ ^133^, ^134^ ^]^ The binding of supramolecular chain segments distinct structural rigidities induces mismatched supramolecular interactions (MMSIs), and the resultant multimers containing equal amounts of 4,4′‐diaminobenzanilide and AD supramolecular segments exhibit both strong binding and structural instable arrangements, wherein the multimers in less‐stable states preferentially slide when subjected to stretching. This is very useful in eliminating excessive supramolecular binding, thus effectively regulating energy dissipation and withstanding external stress. The synthesized elastomer with aromatic amide and ASCZ moieties (SPUU‐DA) exhibits ultrahigh toughness (1.2 GJ·m^−3^), true stress at break (2.3 GPa), and excellent healing ability (Figure 8b).^[^ ^133^ ^]^ Similarly, their team designed another covalently cross‐linked polyurea‐urethane network based on the MMSI strategy (Figure 8c).^[^ ^134^ ^]^ The optimized thermoset elastomer (SPUUN‐IE) containing ASCZ moieties and urea bonds demonstrates unprecedentedly high toughness (1245.2 GJ·m^−3^), ultrahigh ultimate engineering stress (110.8 MPa), robust healing capacity (mechanical properties completely restored after heating at 60 °C for 6 h with the assistance of N,N‐dimethylformamide) and ≈ 100% recycling efficiency after multiple reprocessing. The proposed MMSIs‐based strategy can theoretically guide the rational design of ultra‐strong and ultra‐tough polyureas and other related materials.

### 3.4 Complexes

#### 3.4.1 Polyurea‐Other Polymer Block Copolymers

**Figure 9:**
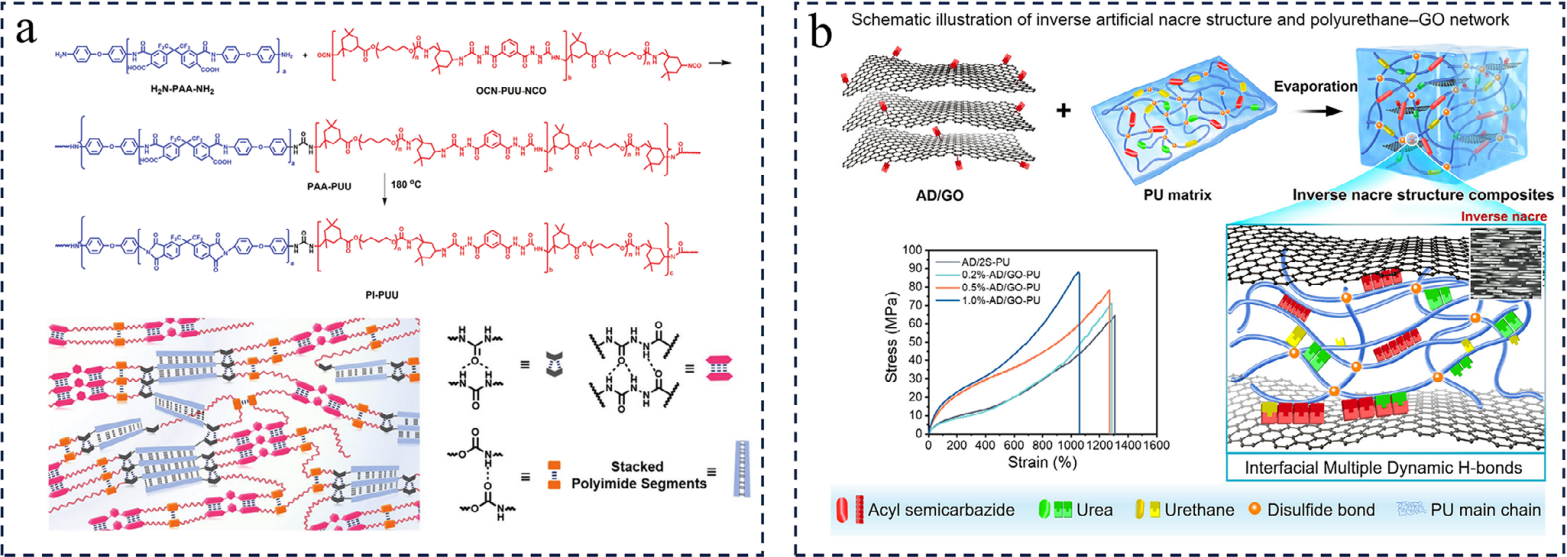
a) Synthetic routes of PI‐PUU. Schematic illustration of the structure of PI‐PUU elastomer. Reproduced with permission.^[^ ^135^ ^]^ Copyright 2023, Wiley. b) Schematic of the PU‐GO network with an inverse artificial nacre structure. Stress‐strain curves of the PU materials. Reproduced with permission.^[^ ^22^ ^]^ Copyright 2022, American Chemical Society.

Well‐designed phase‐separated nanostructures can effectively balance the mechanical strength, stretchability, and elasticity of polyureas while imparting self‐healing property. However, the development of the above self‐healing polyurea materials derived from the polyaddition reaction of isocyanate, conventional soft chain segments (such as PTMEG, PCL), and amine chain extender seems to have reached a bottleneck. Commercial block copolymers such as tri‐blocked poly(styrene‐butadiene‐styrene) have excellent mechanical properties due to the unique two‐phase structure. Inspired by these commercial block copolymers, Sun et al. fabricated ultra‐strong and healable elastomers (PI‐PUU) by copolymerizing rigid polyimide (PI) and polyurea–urethane (PUU) (**Figure** **9**a).^[^ ^135^ ^]^ The PI‐PUU exhibits an ultra‐high tensile strength of ≈142 MPa and an extremely high toughness of ≈527 MJ·m^−3^, and is able to almost completely repair damage efficiently at 50 °C for 24 h with the help of N,N‐dimethylformamide. The superior performance is attributed to the synergistic effect of the rigid PI segments and flexible PUU chain segments containing multiple H‐bonds. Specifically, the PI segments self‐assemble into rigid nanostructures based on π–π interactions and act as nanofillers to significantly enhance the elastomers, while the flexible PUU chain segments are crosslinked with multiple H‐bonds, which can effectively dissipate the strain energy and release the hidden length of the PUU chains, resulting in excellent ductility and toughness of the PI‐PUU elastomers.

#### 3.4.2 Nanofillers‐Reinforced Polyurea Composites

Utilizing the bridging and entanglement effects of nanofillers to strengthen and toughen polyureas is an effective strategy.^[^ ^48^, ^136^, ^137^ ^]^ An urgent issue that needs to be addressed in this type of research is the poor compatibility and inhomogeneous dispersion of nanofillers in the polymer matrix. Besides, in the context of self‐healing polyurea composites, the introduction of rigid particles is quite possible to induce the loss of healing properties due to restricted mobility in the composite. To address the above limitation, surface chemical functionalization of nanofillers is a promising option for the use of nanofillers in high‐performance self‐healing polyurea design.^[^ ^137^, ^138^, ^139^ ^]^ These modified nanofillers are usually able to form strong interfacial interactions with the polyurea matrix in a covalent or non‐covalent manner, which facilitates the stable dispersion of the nanosheets in the polyurea matrix and enhances the strength and toughness of these composite materials. Meanwhile these interactions also act as reversible bonds to ensure the dynamic properties required for self‐healing.^[^ ^48^, ^139^ ^]^ For example, Wang et al. incorporated adipic dihydrazide modified Graphene oxide (AD/GO) nanosheets with multiple H‐bonds into their well‐designed polyurea‐urethane matrix to obtain supramolecular composites with inverse artificial nacre structures (Figure 9b).^[^ ^22^ ^]^ The addition of AD/GO nanosheets can provide abundant dynamic multiple H‐bonds at the interface between nanosheets and matrix, and simultaneously promote the formation of special phase‐separated microstructures in the polyurea‐urethane matrix and the formation of 3D crosslinked networks. Compared with the pure polyurea‐urethane matrix without added nanosheets, the strength of the composites was increased from 64.6 MPa to 78.3 MPa and the toughness from 345.8 MJ·m^−3^ to 505.7 MJ·m^−3^. Due to the appropriate nanosheet content and abundant interfacial H‐bonds, the self‐healing properties of the composites exhibit a negligible decrease compared to pure matrix, and the healing efficiency is as high as 88.6% after contact at 25 °C for 24 h. Similarly, Liu et al. added SiO_2_ nanofillers functionalized with UPy motifs (SiO_2_‐UPy) to their well‐designed polymer matrices containing dynamic borate bonds and quadruple hydrogen bonds.^[^ ^139^ ^]^ The strong interfacial interactions via H‐bonds between SiO_2_‐UPy nanofillers and UPy motifs in the polymer chains enable the composites to display superior strength (60 MPa) and considerable toughness (520 MJ·m^−3^) as well as excellent self‐healing ability. In addition, many functionalized fillers, including MXene,^[^ ^140^ ^]^ WS_2_,^[^ ^137^ ^]^ nanodiamond,^[^ ^141^ ^]^ POSS,^[^ ^142^ ^]^ and MWCNTs,^[^ ^143^ ^]^ have been confirmed to have a better improvement on the comprehensive performance of polyurea, and the constant efforts of researchers are needed to further apply these functionalized fillers to the design of high‐performance self‐healing polyurea in the future.

## 4 Potential Applications

**Figure 10:**
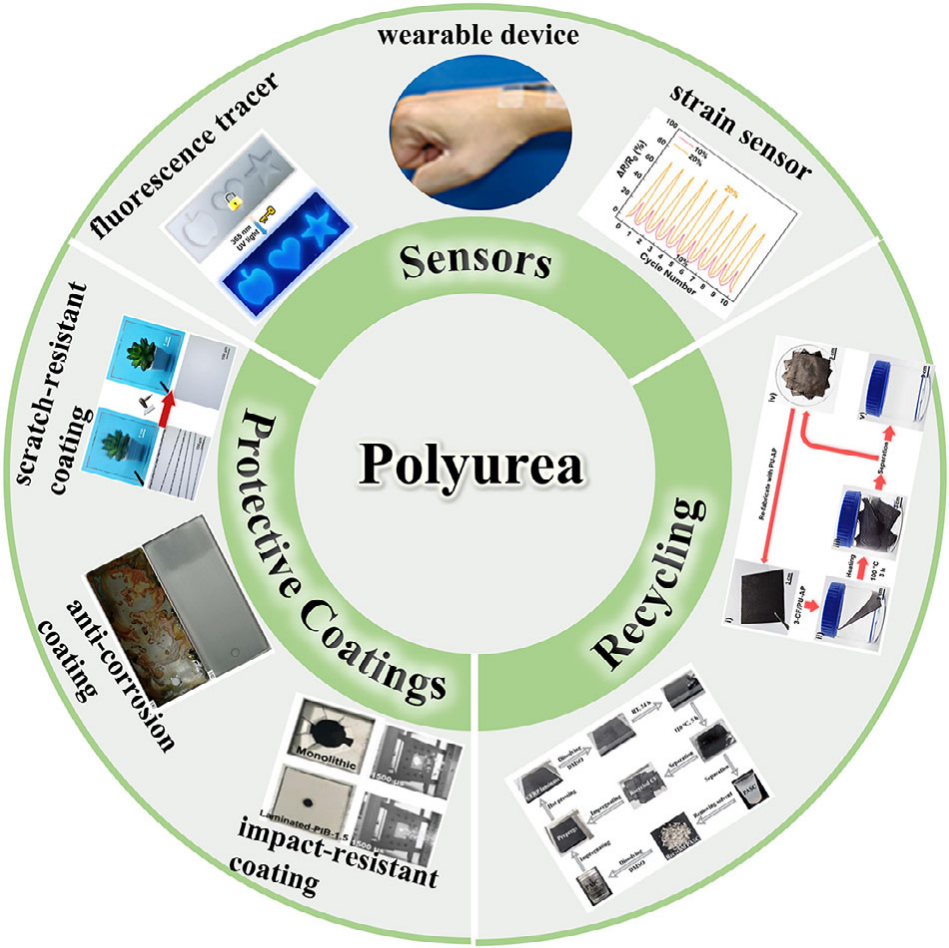
Application areas of mechanically robust self‐healing polyurea.

PUs can be fabricated as plastics, elastomers, fibers, and many other forms depending on their formulation, which gives them the potential to be used in a variety of applications. At present, high‐performance self‐healing polyurea has potential applications in the following areas (**Figure** **10**).

### 4.1 Application in Sensors

For engineering materials, imperceptible mechanical/chemical damage is an important factor in reducing the overall safety and longevity of materials, so the ability to detect material damage is just as important as the damage repair capability. Wu et al. prepared a high‐performance self‐healing polyurea‐urethane elastomer by using a fluorescent chain extender, showing an attractive blue fluorescence under 365 nm UV light, which is attributed to the aggregation‐induced quenching mechanism of fluorescent chain extender.^[^ ^115^ ^]^ Changes in the structure of the elastomer due to mechanical/chemical damage can cause changes in the fluorescence intensity. So not only does elastomer have the ability to self‐repair damage, fluorescent tracers can also act as microcrack detectors and track the healing process, providing early warning of potential failures. Additionally, polyurea‐urethane elastomer solutions are sprayed onto nonfluorescent paper and quickly form films with customizable patterns. Under daylight, these sprayed patterns are indistinguishable, but they exhibit blue fluorescence under UV light at 365 nm. The fluorescent patterns disappear completely when the UV light is turned off. This ability to simulate the process of encrypting and decrypting information makes polyurea elastomers promising for applications as anti‐counterfeiting materials, such as branded anti‐counterfeiting marks on merchandise.

Due to excellent flexibility, mechanical and self‐healing properties, polyureas are often considered ideal substrates for conductive elastomers and flexible wearable sensors. A typical way to prepare such sensors is to embed conductive fillers, such as graphene,^[^ ^144^, ^145^ ^]^ carbon nanotubes,^[^ ^66^, ^146^ ^]^ MXene,^[^ ^147^ ^]^ liquid metal,^[^ ^44^, ^117^ ^]^ ionic liquids,^[^ ^148^ ^]^ etc., into elastomers. For example, Zhang et al. first synthesized an ionic polyurea‐urethane elastomer (EPU‐QDP_3/1_‐SS) with high tensile strength (39.9 ± 4.4 MPa) and high elongation at break (1930 ± 345%), and then obtained a flexible and stretchable strain sensor (EPU‐CNT) by spraying CNTs‐COOH as a conductive filler on the surface of EPU‐QDP_3/1_‐SS.^[^ ^66^ ^]^ The CNTs‐COOH formed ion interactions with the ionic elastomer, which significantly enhances the interfacial adhesion between the two materials and ensures the good self‐healing function of the EPU‐CNT conductive film. The strain sensor based on EPU‐CNT exhibits high sensitivity in a wide sensing range and good stable strain response, which are highly promising as a sensitive self‐healing wearable strain sensor to monitor various human activities. Luo and coworkers prepared a composite strain sensor by combining their polyurea‐urethane material (PUSS‐4), which has excellent mechanical properties and self‐healing function, with CNF and MXene.^[^ ^147^ ^]^ MXene and CNF are uniformly dispersed inside the composite, establishing a stable conductive network and simultaneously optimizing the mechanical properties of the material without compromising its resilience. This composite strain sensor is able to maintain a stable relative resistance change rate across different strain levels, indicating excellent sensing performance and is shown to be useful for monitoring human joint motion. Utilizing the ample hydrogen bond units present in PUSS‐4, the composite sensor exhibits significant damage self‐healing capability.

### 4.2 Protective Coatings

Due to the excellent mechanical properties, good adhesion to the substrate, and convenient construction methods, polyurea is commonly used as a protective coating for a variety of purposes, such as in industry to prevent abrasion and corrosion, in engineering to resist impact damage, and in military/defense for ballistic protection and blast resistance.^[^ ^15^, ^149^, ^150^ ^]^

High‐performance self‐healing polyurea materials can be used as scratch/abrasion‐resistant elastomer coatings to protect substrates.^[^ ^34^, ^151^ ^]^ When the coating is scratched or subjected to abrasion, the coating needs to effectively dissipate the energy concentrated under the scratched area, and the high toughness of polyurea facilitates the dissipation of strain energy. At the same time, the high strength, high elasticity, and strain‐adaptive stiffening of polyurea facilitate resistance to the formation of scratches. Furthermore, the self‐healing property favors the recovery of microcracks once cracks have formed. Sun et al. prepared a scratch‐resistant coating by pouring a solution of their prepared PU‐UPy_0.2_‐DPA_0.8_ elastomer onto a glass substrate.^[^ ^151^ ^]^ The PU coating is highly transparent and no clear scratches can be observed under an optical microscope after 70 scrapings with steel wool at an average pressure of 13.0 kPa. In addition to excellent mechanical properties, polyurea materials are characterized by excellent corrosion resistance, chemical solvent resistance, and weather resistance. Zhang et al. coated their prepared C‐PU on a steel substrate and placed it in a 10% aqueous NaCl solution for 7 d and then exposed it to air for 1 d.^[^ ^152^ ^]^ The result was that the surface of the steel plate without the C‐PU coating was adhered with a dense Fe_2_O_3_ film, while the surface of the C‐PU‐coated steel was clean and smooth to the naked eye. The polyurea composites incorporated with AD/GO nanosheets prepared by Wang et al. exhibited excellent corrosion resistance and gas permeation resistance.^[^ ^22^ ^]^ The corrosion resistance and gas permeation resistance of the polyurea composites increased with the increase in the content of AD/GO nanosheets, which is attributed to the parallel arrangement of AD/GO nanosheets, resulting in a tortuous penetration path for corrosive media and gas molecules. 0.5% AD/GO‐PU composite coating provides excellent protection for metal substrates in water, and it exhibits water insensitivity and is self‐healing in salt water. These materials are promising to make a contribution towards corrosion protection in underwater/marine engineering.

In the field of impact resistance, there is an urgency to deal with the impact of high‐momentum objects against systems or to cushion high‐momentum systems.^[^ ^153^, ^154^ ^]^ Numerous studies have shown that polyurea has a special ability to alter/dispersed shock waves and to absorb the kinetic energy associated with these shock waves under high strain speed loading. This energy‐absorbing capacity is usually related to their ability to undergo a strain‐induced phase transition, during which the material changes from a rubbery state to a glassy state.^[^ ^150^, ^154^ ^]^ High strength, high modulus, and high toughness self‐healing polyurea materials are preferred for cushioning protection. On the one hand, the high strength and modulus facilitate the uniform diffusion of impact momentum from the contact point to the interface, relieving the stress concentration on the protected substrate. On the other hand, the high toughness and dynamic properties are conducive to the absorption and dissipation of impact energy during the deformation process, greatly mitigating the effect of shock waves on the substrate structure.^[^ ^155^ ^]^ Thus, these materials can be used as coatings on the interior/exterior surfaces of structures or as interlayers in composite structures to protect diverse substrates (e.g., concrete, metals, ceramics, glass) by taking advantage of their impact resistance.^[^ ^7^, ^156^, ^157^ ^]^

Wang et al. coated their prepared polyurea on the front side of steel plates, and evaluated the impact resistance of the polyurea materials by drop hammer impact test.^[^ ^23^ ^]^ The uncoated polyurea steel plate developed an obvious circular crater area around the center impact point, with deeper craters and obvious cracks on the backside of the plate, while the craters on the polyurea‐coated steel plate were shallower and no cracks were seen on the backside. They also conducted invasion penetration experiments to investigate the protective properties of polyurea coatings under low‐velocity projectile impact. The post‐target residual velocity reduction of the polyurea‐coated steel plates was significantly greater than that of the uncoated plates, and this effect was more pronounced as the thickness of the polyurea coating increased. One‐component polyurea containing a large number of urea moieties (PIB‐1.5) was composited with alumina ceramic sheets to obtain a novel composite material by Zhang and co‐workers, which exhibiting high modulus, high toughness, and excellent impact resistance.^[^ ^158^ ^]^ Compared with ceramic monolithic structures, epoxy/ceramic composites, polyurea/ceramic composites, and PMMA/ceramic composites, PIB‐1.5/ceramic composites exhibit ultra‐low peak forces on drop hammer impact tests, indicating excellent energy‐dissipation capability. They also performed air gun projectile impact tests at high impact velocities as well as finite element simulations to verify the excellent impact resistance of the PIB‐1.5/ceramic composites. When the projectile contacts the composite material, it produces a large deformation and penetrates the composite material, resulting in the composite structure being intact and leaving a small bullet hole. In contrast, when the projectile contacts the ceramic monolithic structure, it directly penetrates them, resulting in the monolithic structure being broken and leaving a larger bullet hole. This work contributes to new ideas for the design of engineering protective materials for ballistic/impact resistance that can be produced on an industrial scale.

### 4.3 Recycling

The highly dynamic properties in the self‐healing polyurea network give most polyureas the ability to reconstructing network topology and be recyclable through postprocessing techniques such as solvent‐assisted recovery and heat recovery. Polyurea can be used to prepare fiber‐reinforced polymer composites (FRCs) because it is rich in hydrogen bonds and has strong adhesion to fibers.^[^ ^65^ ^]^ It has been demonstrated that FRCs play a vital role in further strengthening the structure against blast loads.^[^ ^159^ ^]^ But the recycling of FRCs is difficult. These high‐performance self‐healing polymers provide a great opportunity to resolve this issue for FRPs. Xia et al. employed their developed polyurea material (a stress at break of 100 MPa and a self‐healing efficiency of 94.4%) as the matrix resin to fabricate the carbon fiber (CF) reinforced polymer composites which exhibited an interlaminar shear strength of 40 MPa and a healing efficiency of 76.2%.^[^ ^132^ ^]^ The highly dynamic nature of the polymer resins allows for a nearly non‐destructive closed recycling of PU/CF composites by means of the solvolytic method. The regenerated PU/CF composites prepared from recycled fibers and resins still maintain good mechanical properties. The self‐healing performance of the PU/CF composite can overcome the delamination problem generated between resin and fiber layers. Sun’ team fabricated a high‐performance CF/PU‐AP composites through complexation of CF cloths and robust PU‐AP elastomer.^[^ ^65^ ^]^ The recycling of CF/PU‐AP composites was realized by heating them in DMAc, cleaving the PU‐AP, and desorbing the PU‐AP from the surface of CF cloths. the CF/PU‐AP composites prepared using recycled CF cloths exhibited the same mechanical properties as those prepared using virgin CF cloths.

## 5 Summary and Outlook

In this review, the major problems existing during the design of mechanically robust self‐healing polyurea are analyzed, which are mainly some trade‐offs between the intrinsic properties, the relationship between polyurea structure and performance is briefly described, and the current strategies adopted to develop mechanically robust self‐healing polyureas are summarized. Moreover, we have listed possible application situations for these polyurea materials. In conclusion, significant progress has been made in the development of self‐healing polyureas with mechanical robustness, but most of the current research is still at the laboratory stage. In order to translate laboratory samples into practical applications, a significant research effort is needed to address several challenges.

1. Considering the nature of the raw materials and the precise control required for the synthesis process, most self‐healing polyureas have been prepared by solvent‐based polymerization method so far. The use of organic solvents in large quantities not only restricts large‐scale production but also poses serious environmental pollution risks. There is an urgent need to develop a solvent‐free, green, and eco‐friendly synthesis strategy suitable for industrial‐scale production.
2. Most existing high‐performance self‐healing polyurea materials require high energy input, such as thermal or UV irradiations, to facilitate damage repair, which is inconvenient for practical applications. Designing polyurea materials that can heal under mild conditions (e.g., ambient temperature, humidity, visible light) is the target of much research. Some of the examples mentioned in this review demonstrate the feasibility of combining self‐healing capability and high robustness at room temperature.^[^ ^42^, ^78^, ^107^ ^]^ However, the rapid rate of bond exchange and chain mobility that allows self‐healing at room temperature typically leads to materials that creep under these same conditions. Therefore, new strategies are needed to achieve the delicate balance between healability, strength, toughness, and creep resistance.
3. For wide‐opened cracks in materials, the first step in damage repair is to bring the two damaged surfaces into close contact, which often requires the assistance of external forces. Considering the need for some practical applications, there is an urgent need to develop polyurea materials that can close the cracks automatically. Shape memory polymers have the ability to “memorize” permanent shapes; they could be controlled to maintain temporary shapes and return to the memorized shape under appropriate conditions triggered by stimuli such as heat, Ph, light, deformation, and so on.^[^ ^160^, ^161^ ^]^ Recently, shape memory assisted self‐healing strategy has been extensively investigated to repair cracks in polymers by using the shape memory effect to make the surface crack of the polymers intimate contact, thus achieving damage healing through the dynamic nature of the polymer network without external force. The incorporation of shape memory into a tough polyurea network remains to be further investigated.^[^ ^27^, ^46^, ^162^ ^]^
4. Toughening and strengthening within self‐healing polyurea networks is a multicomponent synergistic process, and it remains a challenging problem to analyze it more accurately and even quantitatively due to the limitations of existing technological levels. There is an urgent need for qualitative and quantitative analyses of events at the molecular level, as well as secondary structures, in order to establish their effects on structure‐property relationships of final polyureas as a function of design parameters such as molecular structure of soft and hard segments. In addition to the development of more advanced means of instrumental characterization, computer simulation, and modeling have been useful in answering many perplexing questions that are not readily measurable experimentally, especially for predicting the properties of polyurea materials. Sets of solutions with broad applicability in this field are awaiting continuous contributions from theoretical and experimental scientists.

## Conflict of Interest

The authors declare no conflict of interest.
